# Lorentz skew scattering nonreciprocal magneto-transport

**DOI:** 10.1038/s41467-026-71789-y

**Published:** 2026-05-08

**Authors:** Xiu Fang Lu, Xue-Jin Zhang, Naizhou Wang, Jin Cao, Dan Zhao, Hui Wang, Tao Wu, Xianhui Chen, Shen Lai, Shuigang Xu, Cong Xiao, Shengyuan A. Yang, Weibo Gao

**Affiliations:** 1 Hangzhou International Innovation Institute, Beihang University, Hangzhou, China; 2 Institute of Applied Physics and Materials Engineering, Faculty of Science and Technology, University of Macau, Macau SAR, China; 3 Department of Physics, School of Science, Westlake University, Hangzhou, China; 4 Department of Physics and Hefei National Laboratory for Physical Science at Microscale, University of Science and Technology of China, Hefei, Anhui China; 5 Division of Physics and Applied Physics, School of Physical and Mathematical Sciences, Nanyang Technological University, Singapore, Singapore; 6 Interdisciplinary Center for Theoretical Physics and Information Sciences (ICTPIS), Fudan University, Shanghai, China; 7 Research Laboratory for Quantum Materials, Department of Applied Physics, The Hong Kong Polytechnic University, Hong Kong, China; 8 School of Electrical and Electronic Engineering, Nanyang Technological University, Singapore, Singapore; 9 Centre for Quantum Technologies, Nanyang Technological University, Singapore, Singapore; 10 National Centre for Advanced Integrated Photonics (NCAIP) Singapore, Nanyang Technological University, Singapore, Singapore

**Keywords:** Electronic properties and materials, Topological matter, Electronic and spintronic devices

## Abstract

In materials with broken inversion symmetry, nonreciprocal magneto-transport manifests as a bilinear dependence of charge conductivity on electric and magnetic fields. This phenomenon is rooted in symmetry and electronic quantum geometry and is relevant for rectification and detection technologies. Experimental studies generally attribute nonreciprocal magneto-transport to Zeeman-driven mechanisms and exhibit quadratic scaling with conductivity. Here, we report a microscopic mechanism based on Lorentz skew scattering in BiTeBr, arising from the cooperation of classical Lorentz force and quantum skew scattering, exhibiting a quartic scaling of the nonreciprocal response. Systematic measurements on samples with different mobilities reveal a crossover between Zeeman-related and Lorentz-skew scattering-dominated regimes, uncovering the mobility plays a central role in determining the dominant mechanism. Our finding unveils the leading mechanism in high-mobility systems and suggests a universal principle towards strong nonreciprocal response by enhancing electronic relaxation time in topological materials, rendering guidance for low-dissipation rectifiers and high-performance quantum electronics.

## Abstract (web summary)

Nonreciprocal magneto-transport is often attributed to Zeeman-driven mechanisms. Here, the authors identify Lorentz skew scattering nonreciprocal magneto-transport in BiTeBr which emerges from the cooperation of classical Lorentz force and quantum skew scattering.

## Introduction

In terms of current response, nonreciprocal magneto-transport (NRMT) corresponds to the nonreciprocal component ${j}^{NR{MT}}=\chi {E}^{2}B$^1–18^. It is nonreciprocal, since it leads to a difference in response when the field (or current) direction is reversed. Such a phenomenon was first studied in materials with chiral structures and was known as electrical magneto-chiral anisotropy^1,19^. Lately, it was actively explored also in achiral systems lacking inversion symmetry^14,20–25^, such as surfaces and interfaces of bulk crystals with central asymmetric lattices; and it came under the names of bilinear magnetoelectric resistance (for longitudinal response, ${j||E}$) or nonlinear planar Hall effect (for transverse response, $j\perp E$). In these studies, the magnetic field is typically applied within the transport plane, such that characteristic features of the response tensor $\chi$ may be extracted from angular dependence of the in-plane magnetic field. It was found that the NRMT often manifests intriguing quantum geometrical properties of Bloch electrons^2,18,26^. In Weyl semimetals, NRMT may arise from chiral anomaly^27^ or Berry curvature mechanisms^28^. NRMT results detected in other systems were usually attributed to chiral spin textures due to spin-orbit coupling, and the effect of magnetic field is to deform the Fermi surface via Zeeman coupling to magnetic moment of electrons^3–8,29–31^. The experimentally reported NRMT so far is generally weak, and existing understanding of microscopic mechanisms for NRMT is far from complete, both hindering its potential applications.

**Fig. 1:**
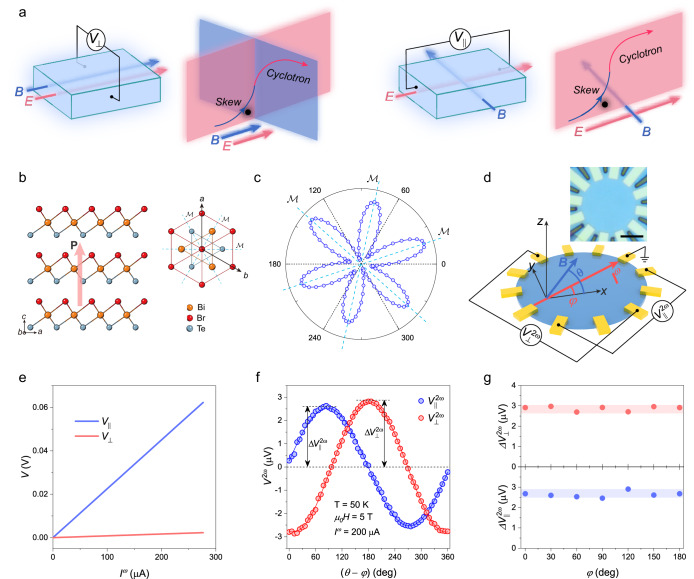
Schematics of Lorentz skew scattering (LSK), the symmetry of BiTeBr crystal structure, and the measurement of nonreciprocal magneto-transport (NRMT). **a** Schematics of the microscopic processes of LSK. Left, transverse LSK transport in parallel fields; right, longitudinal LSK transport in perpendicular fields. **b** Side and top views of the BiTeBr crystal structure. Spontaneous electric polarisation along *c*-axis (pink arrow) arises from inversion symmetry breaking. The blue dashed line (M) denotes the mirror line. **c** The angle-dependent optical second harmonic generation (SHG) of an exfoliated BiTeBr flake. The direction of minimum intensity is identified as the crystal axis. **d** Measurement configuration for NRMT. The *x, y, z*-axes denote the crystal axis, transverse in-plane direction and out-of-plane direction. An alternating current (${I}^{\omega }$) is applied at an angle *φ* to the *x*-axis, while an in-plane magnetic field *B* makes an angle *θ* with the *x*-axis. The second harmonic response is measured simultaneously in the longitudinal (${V}_{{||}}^{2\omega }$) and transverse (${V}_{\perp }^{2\omega }$) directions. Insert: optical image of Device #1. scale bar: 5 µm. **e** The linear longitudinal and transverse voltages, ${V}_{{||}}$ and ${V}_{\perp }$, as a function of ${I}^{\omega }$ at 50 K under zero magnetic field.** f** Nonlinear responses ${V}_{\perp }^{2\omega }$ and ${V}_{{||}}^{2\omega }$ as a function of angle (*θ* – *φ*) measured in the configuration shown in (**d**). ${\Delta V}_{\perp }^{2\omega }$ and ${\Delta V}_{{||}}^{2\omega }$ denote the amplitudes of the NRMT response at transverse and longitudinal direction. **g** ${\Delta V}_{\perp }^{2\omega }$ and ${\Delta V}_{{||}}^{2\omega }$ as a function of the driving current orientation *φ*. Red and blue shading guide the eye, highlighting that ${\Delta V}_{\perp }^{2\omega }$ and ${\Delta V}_{{||}}^{2\omega }$ remain nearly constant.

A common approach to distinguish different mechanisms of nonlinear transport effects is to perform scaling analysis, which examines how the response tensor $\chi$ depends on the longitudinal conductivity ${\sigma }_{{xx}}$^32–48^. Experimentally, this is usually achieved by varying the temperature of measurement, and the resulting scaling relation usually takes a polynomial form: $\chi={\sum }_{i={{\mathrm{0,1,2}}},\ldots }{c}_{i}{\sigma }_{{xx}}^{i}$. As for NRMT in nonmagnetic systems, the Hall response may have an intrinsic contribution with $i=0$^16,49^; whereas for longitudinal response, the previously proposed mechanisms, including the Zeeman-coupling mechanism, is invariably dominated by the $\chi \propto {\sigma }_{{xx}}^{2}$ ${{{\rm{scaling}}}}$^3–8,29–31^. Interestingly, a recent theory proposed a new mechanism for NRMT - the Lorentz skew scattering (LSK)^50^, the schematics of which is shown in Fig. 1a. Distinct from other mechanisms, in LSK, the magnetic field enters via Lorentz force, rather than Zeeman coupling^4–8,15,31^ or other quantum mechanical effects^21,27,28^, which directly affects real-space motion of electrons instead of modifying momentum-space band structures. In the meantime, the quantum geometry of Bloch electrons is encoded in the skew scattering process, whose scattering rate is shown to be related to the Berry curvature on Fermi surface^50^. Most importantly, LSK gives a quartic scaling behaviour $\propto {\sigma }_{{xx}}^{4}$, sharply distinct from all previous NRMT mechanisms. It is shown to be the leading degree term in the scaling relation, which should dominate the NRMT response in systems with high mobility. Since low-dissipation electronic devices commonly desire high-mobility active materials, the LSK mechanism offers a new strategy to enhance NRMT (and rectification) effect for applications. Nevertheless, till now, such distinctive quartic scaling behaviour associated to LSK has not been reported in experiment yet.

In this work, we investigate nonreciprocal magneto-transport in the high mobility *n*-doped polar semiconductor BiTeBr, a 3D system in which the Lorentz force can take effect in the planar setup. The scaling analysis reveals a quartic dependence of the nonreciprocal response on longitudinal conductivity, consistent with the behaviour expected for LSK mechanism. Our experimental result further finds quantitative agreement with theoretical calculations, confirming LSK as the dominant mechanism of the observed nonlinear response. Moreover, by carrying out the systematic transport measurements across multiple samples with different mobilities, we reveal the crossover behaviour of NRMT scaling from Zeeman-coupling to LSK mechanism, uncovering the mobility as a key factor governing the dominant mechanism of NRMT.

## Results and discussion

As shown in Fig. 1b, BiTeBr exhibits threefold rotational symmetry within *ab*-plane and a polar axis along the *c*-axis. The ABC stacking of Te, Bi and Br triangular atomic layers breaks both the inversion symmetry and the horizontal mirror symmetry, which fulfills the requirement of NRMT with in-plane magnetic field. To determine the crystal axes of our BiTeBr flake, we employed the optical second harmonic generation (SHG) technique. As depicted in Fig. 1c, the SHG intensity displays a distinct threefold rotational symmetry, with the direction of minimum (maximum) intensity corresponding to the crystal axis marked as $x$ ($y$). Here, $y$ is the direction within the vertical mirror planes. After determining the crystal axis, we fabricated a high quality, few layer BiTeBr device with the thickness of about 15 nm and 12 radically distributed electrodes (Device #1) (see “Methods” for device details). Our initial step involved assessing the contact quality of these electrodes (See supplementary Fig. S1 for the two-terminal *I-V* curves for all 12 electrodes), which demonstrated good contact across all electrodes. We then carried out the basic characterisation on Device #1 (Supplementary Fig. S2). The electron carrier density obtained from Hall measurement is $\sim 10\times {10}^{18}{{{\rm{c}}}}{{{{\rm{m}}}}}^{-3}$, which is similar to previous studies^4^. However, the resistivity ${\rho }_{{xx}}$ is smaller than previously reported values (varying from 0.30 mΩcm at 2 K to ∼1.28 mΩcm at 300 K). This reduced resistivity indicates a higher mobility of ~2000 cm^2^V^−1^s^−1^ at 2 K for our sample, substantially exceeding the previously reported value of 295 cm^2^V^−1^s^−1^ at 2K^4,46^, suggesting improved quality in our device.

To measure the linear and nonlinear electrical transport, an AC current (${I}^{\omega }$) at a fixed frequency *ω* = 17.777 Hz was applied in the *ab*-plane of the device, which makes an angle $\varphi$ with respect to the crystal $x$ axis (Fig. 1d). We first characterised the first harmonic voltage drops at longitudinal (${V}_{{||}}$) and transverse (${V}_{\perp }$) directions under zero magnetic field, as shown in Fig. 1e ($\varphi=0$). Both ${V}_{{||}}$ and ${V}_{\perp }$ increase linearly with current, and the magnitude of ${V}_{\perp }$ remains negligible compared to ${V}_{{||}}$, indicating good contact and proper alignment of electrodes. Next, we measured the NRMT response of Device #1. The measurement configuration is illustrated in Fig. 1d. We applied a magnetic field within the *ab*-plane of BiTeBr, whose direction described by the polar angle *θ* may vary in plane (details of the electrical measurement shown in Methods). We then measured the second-harmonic response in both longitudinal (${V}_{{||}}^{2\omega }$) and transverse (${V}_{\perp }^{2\omega }$) directions. Figure 1f shows the results of Device #1 as a function of the relative angle (θ−φ), with a 5 T in-plane magnetic field and a 200 µA driving current at 50 K. To obtain the response signal induced by magnetic field, we subtracted the field-independent background of ${V}_{{||}}^{2\omega }$ and ${V}_{\perp }^{2\omega }$ (Supplementary Fig. S3). As depicted in Fig. 1f, ${V}_{{||}}^{2\omega }$ and ${V}_{\perp }^{2\omega }$ exhibits sine and cosine dependence with a 90° phase shift. Additionally, both ${V}_{{||}}^{2\omega }$ and ${V}_{\perp }^{2\omega }$ flip signs when reversing the direction of the magnetic field. These behaviors are fully consistent with previously reported NRMT characteristics^4,7,51,52^. To characterise the magnitude of response, we use the notation $\Delta {V}_{\perp }^{2\omega }$ ($\Delta {V}_{{||}}^{2\omega }$) to represent the amplitude of the oscillating curve of ${V}_{\perp }^{2\omega }$ (${V}_{{||}}^{2\omega }$). When we changed the orientation of driving current direction, the waveforms of ${V}_{\perp }^{2\omega }$ and ${V}_{{||}}^{2\omega }$ remain invariant as functions of ($\theta -\varphi$) (Supplementary Fig. S4), and the amplitudes, $\Delta {V}_{\perp }^{2\omega }$ and $\Delta {V}_{{||}}^{2\omega }$, remain nearly constant, as presented in Fig. 1g.

**Fig. 2:**
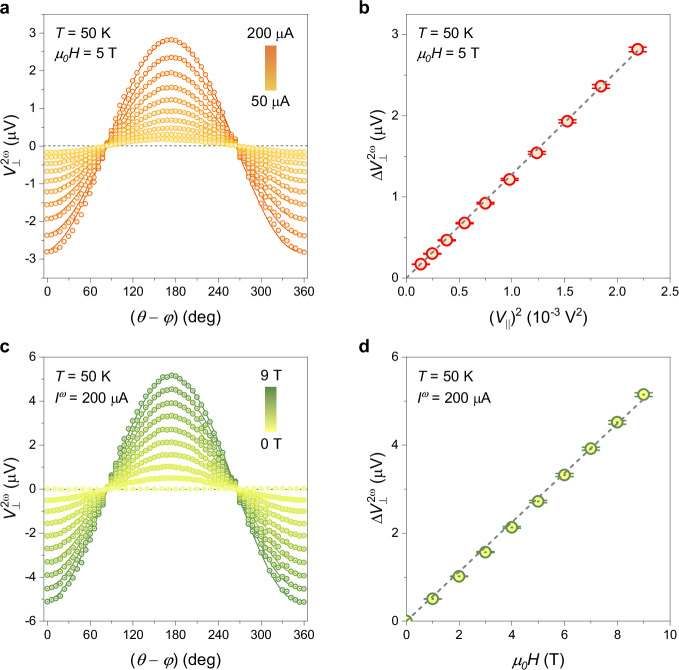
Nonreciprocal magneto-transport in the transverse direction under varying electric and magnetic fields. **a**, **b** Nonlinear transverse response ${V}_{\perp }^{2\omega }$ as a function of angle (*θ* – *φ*) measured with driving current ${I}^{\omega }$ from 50 to 200 µA (**a**), and the corresponding sinusoidal-fit amplitude $\Delta {V}_{\perp }^{2\omega }$ as a function of the square of the linear longitudinal voltage ${V}_{{||}}$ (**b**). **c**, **d** ${V}_{\perp }^{2\omega }$ as a function of angle (*θ* – *φ*) measured under in-plane magnetic field ${\mu }_{0}H$ from 1 to 9 T (**c**), and the corresponding sinusoidal-fit amplitude $\Delta {V}_{\perp }^{2\omega }$ as a function of ${\mu }_{0}H$ (**d**). Error bars in (**b**) and (**d**) represent the standard errors from the sinusoidal fits.

At a fixed current direction, we measured the ${V}_{{||}}^{2\omega }$ and ${V}_{\perp }^{2\omega }$ at 50 K under different magnitudes of driving current and magnetic field, as shown in Fig. 2 (the data of ${V}_{{||}}^{2\omega }$ are shown in Supplementary Fig. S5). One can see that $\Delta {V}_{\perp }^{2\omega }$ increases with ${I}^{\omega }$ (Fig. 2a), and scales with the square of the linear longitudinal voltage ${V}_{{||}}$ (Fig. 2b), i.e., $\Delta {V}_{\perp }^{2\omega }$ $\propto$ ${({V}_{{||}})}^{2}$. Furthermore, for a fixed ${I}^{\omega }$, $\Delta {V}_{\perp }^{2\omega }$ increases linearly with the magnetic field up to at least 9 T (Fig. 2c, d), showing $\Delta {V}_{\perp }^{2\omega }\propto B$. Similar results are also observed in the longitudinal response (Supplementary Fig. S5). These results unambiguously demonstrate that the observed nonlinear signals $\Delta {V}_{\perp }^{2\omega }$ and $\Delta {V}_{{||}}^{2\omega }$ correspond to the NRMT response, which is $\propto {E}^{2}B$.

This identification of measured signal with NRMT also explains the observed angular dependence of ${V}_{{||}}^{2\omega }$ and ${V}_{\perp }^{2\omega }$. Constrained by the ${C}_{3v}$ point group symmetry of BiTeBr, the longitudinal and transverse NRMT responses have angular dependence in the outer product form $\sim {{{\bf{I}}}}\times {{{\bf{B}}}}{{{\boldsymbol{\cdot }}}}\hat{{{{\bf{P}}}}}$ and inner product form ${{{\boldsymbol{\sim }}}}{{{\bf{I}}}}\cdot {{{\bf{B}}}}$, respectively (see “Methods”). Here, $\hat{{{{\bf{P}}}}}$ is the unit vector along the polar axis (*z*-axis). This accounts for the observed sine and cosine dependence: ${V}_{{||}}^{2\omega }\sim \Delta {V}_{{||}}^{2\omega }\sin \left({{{\rm{\theta }}}}-\varphi \right)$ and ${V}_{\perp }^{2\omega }\sim \Delta {V}_{\perp }^{2\omega }\cos (\theta -\varphi )$. The ${C}_{3v}$ symmetry ensures that the amplitudes $\Delta {V}_{\perp }^{2\omega }$ and $\Delta {V}_{{||}}^{2\omega }$ remains nearly constant regardless of the current injection direction. Such a clean angular dependence on the relative angle of the driving current and magnetic field is unique to in-plane field, which also helps exclude the possibility of out-of-plane field component due to misalignment.

**Fig. 3:**
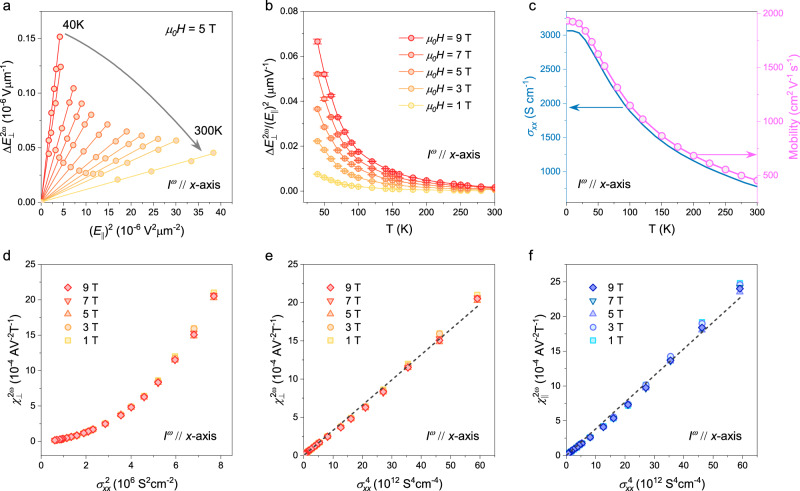
Temperature dependence and scaling law of the nonreciprocal magneto-transport observed in BiTeBr Device #1. **a** $\Delta {E}_{\perp }^{2\omega }$ as a function of ${({E}_{{||}})}^{2}$ at temperatures from 40 K to 300 K, measured under a 5 T in-plane magnetic field with ${I}^{\omega }$ applied along *x*-axis. The slope of $\Delta {E}_{\perp }^{2\omega }$ - ${({E}_{{||}})}^{2}$ decreases with increasing temperature. **b** $\Delta {E}_{\perp }^{2\omega }/{({E}_{{||}})}^{2}$ as a function of temperature, measured under magnetic fields from 1 to 9 T. Error bars represent the standard errors of $\Delta {E}_{\perp }^{2\omega }/{({E}_{{||}})}^{2}$ obtained from linear fits to $\Delta {E}_{\perp }^{2\omega }$ - ${({E}_{{||}})}^{2}$. **c** Temperature dependence of longitudinal conductivity ${\sigma }_{{xx}}$ (left) and mobility (right). **d** Scaling the NRMT coefficient ${\chi }_{\perp }^{2\omega }$ as ${\sigma }_{{xx}}^{2}$. **e**, **f** NRMT coefficient ${\chi }_{\perp }^{2\omega }$ (**e**) and ${\chi }_{\Vert }^{2\omega }$ (**f**) as a function of ${\sigma }_{{xx}}^{4}$, showing a linear dependence. Dashed lines indicate linear fits.

Having identified the NRMT response, we next investigated the underlying physical mechanism by the scaling analysis. To perform this, we measured ${V}_{\perp }^{2\omega }$ and ${V}_{{||}}^{2\omega }$ across varying magnitudes of electric and magnetic fields at different temperatures. Figure 3a shows the extracted nonlinear transverse response field $\Delta {E}_{\perp }^{2\omega }$ as a function of longitudinal electric field ${E}_{{||}}$ at different temperatures under a 5 T in-plane magnetic field. Here, ${E}_{{||}}\equiv {V}_{{||}}/{L}_{{||}}$ and $\Delta {E}_{\perp }^{2\omega }\equiv \Delta {V}_{\perp }^{2\omega }/{L}_{\perp }$, with ${L}_{\perp }$ and ${L}_{{||}}$ being the transverse and longitudinal lengths of the device, respectively. As depicted in Fig. 3a, $\Delta {E}_{\perp }^{2\omega }$ scales linearly with ${({E}_{{||}})}^{2}$ at all temperatures, and the slopes of $\Delta {E}_{\perp }^{2\omega }$ - ${({E}_{{||}})}^{2}$ curves decrease monotonically with increasing temperature. In addition to the 5 T data, we also measured $\Delta {E}_{\perp }^{2\omega }$ - ${({E}_{{||}})}^{2}$ under varying magnetic fields from 1 T to 9 T, which exhibit the same features. We plot all the measured values of $\Delta {E}_{\perp }^{2\omega }/{({E}_{{||}})}^{2}$ in Fig. 3b, which shows a consistent decrease with temperature across all magnetic fields. The longitudinal response $\Delta {E}_{{||}}^{2\omega }$ exhibits similar behaviours, as shown in Supplementary Fig. S6.

For scaling analysis, we focus on the NRMT response coefficient ${\chi }_{\perp }^{2\omega }=\sigma \frac{\Delta {E}_{\perp }^{2\omega }}{{({E}_{{||}})}^{2}B}$ and ${\chi }_{{||}}^{2\omega }=\sigma \frac{\Delta {E}_{{||}}^{2\omega }}{{({E}_{{||}})}^{2}B}$. Relating them to the rank-4 NRMT response tensor defined by ${j}_{i}^{2\omega }={\chi }_{{ijk}\ell }^{2\omega }{E}_{j}^{\omega }{E}_{k}^{\omega }{B}_{\ell }$, where all indices $\in \{x,y\}$ and repeated indices are summed over, one finds that ${\chi }_{\perp }^{2\omega }=|{\chi }_{{yxxx}}^{2\omega }|$ and ${\chi }_{{||}}^{2\omega }=\left|{\chi }_{{xxxy}}^{2\omega }\right|$ in our setup. As discussed, previously reported mechanisms for NRMT give a $\chi \propto {\sigma }_{{xx}}^{2}$ scaling^3–6,8,31^. The ${\sigma }_{{xx}}$ as a function of temperature is presented in Fig. 3c (due to ${C}_{3v}$ symmetry, the linear conductivity is isotropic in the plane). We plot ${\chi }_{\perp }^{2\omega }$ (Fig. 3d) and ${\chi }_{{||}}^{2\omega }$ (Fig. S6d) versus ${\sigma }_{{xx}}^{2}$. One can see clearly that the NRMT does not conform to the quadratic scaling relation for conventional mechanisms. To further examine the scaling behaviour of ${\chi }_{\perp }^{2\omega }$ and ${\chi }_{\Vert }^{2\omega }$ with longitudinal conductivity, we plot both quantities as functions of ${\sigma }_{{xx}}^{n}$ (with n = 1 to 4) (Figure S8). As $n$ increases, the dependence of ${\chi }_{\perp }^{2\omega }$ and ${\chi }_{\Vert }^{2\omega }$ becomes progressively more linear. We also present logarithmic plots of ${\chi }_{\perp }^{2\omega }$ and ${\chi }_{\Vert }^{2\omega }$ versus ${\sigma }_{{xx}}$ in Figure S7. The extracted exponents are 3.93 and 4.01, respectively, consistent with a quartic scaling relationship. These results show that the quartic contribution dominates the NRMT response in BiTeBr Device #1, which has not been observed before. This indicates some new mechanism must be in action here.

By carefully examining the scaling behaviour of the data (See Supplementary Fig. S7-S10), we find the curves can be well fitted by a simple quartic scaling relation $\chi={A}_{4}{\sigma }_{{xx}}^{4}$, with vanishingly small intercepts, as shown in Figs. 3e, f. This clean result signifies the overwhelming dominance of the quartic scaling contribution in both the transverse and longitudinal NRMT measured here. Moreover, the magnitudes of the two responses are quite similar, with coefficient ${A}_{4}$ being $0.33\times {10}^{-16}{{{\rm{A}}}}{{{{\rm{V}}}}}^{-2}{{{{\rm{T}}}}}^{-1}{{{{\rm{S}}}}}^{-4}{{{\rm{c}}}}{{{{\rm{m}}}}}^{4}$ for ${\chi }_{\perp }^{2\omega }$, and $0.38\times {10}^{-16}{{{\rm{A}}}}{{{{\rm{V}}}}}^{-2}{{{{\rm{T}}}}}^{-1}{{{{\rm{S}}}}}^{-4}{{{\rm{c}}}}{{{{\rm{m}}}}}^{4}$ for ${\chi }_{{||}}^{2\omega }$. Since the crystal symmetry does not require these values to coincide, the common quartic scaling behaviour and the close magnitudes suggest that the observed transverse and longitudinal nonreciprocal transport should share the same physical origin.

To explore the physical origin of the unconventional quartic scaling behaviour, we first ascertained that the temperature variation of longitudinal conductivity comes from the variation in electronic relaxation time *τ*, i.e., the mobility instead of the carrier density. We measured the Hall resistivity (under perpendicular magnetic fields) and extracted the carrier density of BiTeBr Device #1 across a temperature range from 2 to 300 K (Supplementary Fig. S2). The linearity of Hall resistivity indicates a single band (single-$\tau$) dominated transport in our BiTeBr device (which is also confirmed by our first-principles result). The carrier density varies by 6.5% between 40 K and 300 K, indicating that the temperature dependence of ${\sigma }_{{xx}}$ is primarily governed by changes in $\tau$. (Indeed, as shown in Fig. 3c, the mobility and ${\sigma }_{{xx}}$ exhibit nearly the same temperature dependence.) Therefore, the quartic scaling behaviour in Fig. 3e, f further shows that ${\chi }_{\perp }^{2\omega }\propto {\tau }^{4}$ and ${\chi }_{{||}}^{2\omega }\propto {\tau }^{4}$ regarding the temperature dependence.

We further performed independent measurements and scaling analyses on Device #4 and Device #5, with thicknesses of ~104 nm and ~116.5 nm, respectively. For Device #4 (mobility ~2064 cm^2^V^−1^s^−1^ at 2 K), we analyzed the scaling behaviour following the same procedure as for Device #1 (Figure S11) and found that the ${\sigma }_{{xx}}^{4}$ term is the dominant contribution. For Device #5 (Mobility ~ 1931 cm^2^V^−1^s^−1^ at 2 K), the results are summarised in Figure. S12. Since the longitudinal (${\chi }_{\Vert }^{2\omega }$) and transverse (${\chi }_{\perp }^{2\omega }$) NRMT signals are nearly identical, only ${\chi }_{\Vert }^{2\omega }$ is shown. Again, the NRMT response is primarily governed by the ${\sigma }_{{xx}}^{4}$ term. For Device #4 and Device #5, the carrier density varies by 4.6% and 7.4% in the temperature regime of transport measurement, respectively (Supplementary Table S2). Together, these measurements confirm that the quartic-scaling NRMT ${\chi }^{2\omega }\propto {\tau }^{4}$ are robust and reproducible across different devices.

The observed quartic scaling $\chi \propto {\tau }^{4}$ is consistent with the LSK mechanism. As shown in Ref. ^50^, the LSK gives a NRMT current expressed as ${{{{\bf{j}}}}}^{{{{\rm{LSK}}}}}=-e{\sum }_{l}{g}_{l}^{{{{\rm{LSK}}}}}{{{{\boldsymbol{v}}}}}_{l}$, where ${{{{\boldsymbol{v}}}}}_{l}$ is the velocity of a Bloch electron in state $l$, and

1 $${g}_{l}^{{{{\rm{LSK}}}}}=-{\tau }^{4}\left[{\hat{D}}_{E}\left\{{\hat{D}}_{B},{\hat{I}}_{{sk}}\right\}+{\hat{D}}_{B}\left\{{\hat{I}}_{{sk}},{\hat{D}}_{E}\right\}+{\hat{I}}_{{sk}}\left\{{\hat{D}}_{E},{\hat{D}}_{B}\right\}\right]{\hat{D}}_{E}{f}^{0}$$

is the LSK off-equilibrium distribution function. Here, ${f}^{0}$ is the Fermi distribution, ${\hat{I}}_{{sk}}$ is the skew-scattering collision-integral operator, whose action on a distribution function ${f}_{l}$ is given by ${\hat{I}}_{{sk}}{f}_{l}=-{\sum }_{l^{\prime} }{\omega }_{l^{\prime} l}^{3a}({f}_{l}+{f}_{l^{\prime} })$, with ${\omega }_{l^{\prime} l}^{3a}$ being the skew scattering rate (details in Methods). ${\hat{D}}_{E}=-e{{{\bf{E}}}}\cdot {\partial }_{{{{\boldsymbol{k}}}}}$ and ${\hat{D}}_{B}=-e{{{{\boldsymbol{v}}}}}_{l}\times {{{\bf{B}}}}\cdot {\partial }_{{{{\boldsymbol{k}}}}}$ are differential operators corresponding to driving terms of electric force and Lorentz force in the Boltzmann kinetic equation, and $\left\{{\hat{D}}_{E}{,\hat{D}}_{B}\right\}\equiv {\hat{D}}_{E}{\hat{D}}_{B}+{\hat{D}}_{B}{\hat{D}}_{E}$. In the presence of both impurity and phonon scattering, the relaxation time $\tau$ is contributed by both, and ${g}_{l}^{{{{\rm{LSK}}}}}\propto {\tau }^{4}$ leads to the quartic scaling behaviour of the nonreciprocal transport. It was shown that the LSK mechanism gives the highest degree term in the scaling relation^50^, so it is expected to dominate the response for high-mobility samples, which is the case here. As shown in Fig. 3c, the mobility rises from 500 $c{m}^{2}{V}^{-1}{s}^{-1}$ at 300 K to about 2000 $c{m}^{2}{V}^{-1}{s}^{-1}$ at 2 K. We note that in linear anomalous transport, it has been well established that the skew scattering contribution dominates over other contributions in 3 d transition metals Fe, Co, and Ni when ${\sigma }_{{xx}}\ge {10}^{6}{\Omega }^{-1}c{m}^{-1}$, which is termed as high conductivity regime^53^. Since the carrier densities of these metals are about ${10}^{23}c{m}^{-1}$, the mobilities of these high conductivity systems are smaller than 100 $c{m}^{2}{V}^{-1}{s}^{-1}$. Therefore, the mobility in our BiTeBr Device#1 can indeed be viewed as high in NRMT, benefitting the observation of LSK transport. Moreover, other BiTeBr devices possessing mobilities of similar magnitudes also exhibit the quartic scaling characteristic of LSK transport (Device#4 and #5, Supplementary Fig. S11 and S12).

**Fig. 4:**
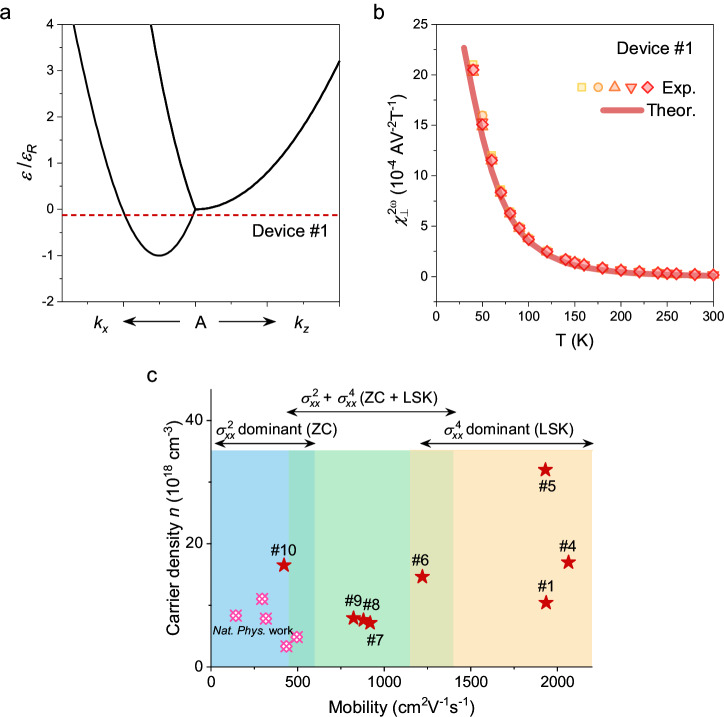
The LSK as the microscopic physical origin of the observed NRMT in high-mobility BiTeBr. **a** Band structure of the Rashba model fitted from the first-principles bands of BiTeBr. The red dashed line represents the Fermi level of Device#1. Here ${\varepsilon }_{R}=\frac{{\alpha }_{R}^{2}}{4t}=42$ meV is the Rashba energy. **b** The NRMT coefficient ${\chi }_{\perp }^{2\omega }$ of Device#1. The scatters represent experimental data, whereas the curves depict theoretical results of LSK effect. **c** Crossover behaviour of dominant ${\sigma }_{{xx}}$-scaling with the device mobility. Here, LSK and ZC denote the Lorentz skew scattering and Zeeman coupling mechanisms, respectively.  represent the devices measured in our work;  represent data on NRMT in BiTeBr extracted from Ref. ^4^.

To perform quantitative evaluation based on the above theoretical formula, we first calculate the band structures of BiTeBr by first-principles calculation (Supplementary Fig. S18). The result shows that the low-energy physics is well described by the following 3D Rashba model around $A$ point of the Brillouin zone:

2 $${{{\mathcal{H}}}}=\frac{1}{2{m}_{{xy}}}\left({k}_{x}^{2}+{k}_{y}^{2}\right)+\frac{{k}_{z}^{2}}{2{m}_{z}}+{\alpha }_{R}\left({k}_{y}{\sigma }_{x}-{k}_{x}{\sigma }_{y}\right)+\frac{1}{2}\left({k}_{+}^{3}+{k}_{-}^{3}\right)\left(\lambda {\sigma }_{z}+{\lambda }_{0}{k}_{z}\right),$$

where the first two terms are usual quadratic dispersing terms, the third term is a Rashba-type spin-orbit coupling, and the last term is a warping term which ensures the model recovers the ${C}_{3v}$ symmetry of the system. The model parameters are obtained by fitting the first-principles band structure (see “Methods”). This 3D Rashba model features a Weyl point at $k=0$, i.e., the *A* point. The Fermi level position estimated from the carrier density value is quite close to the Weyl point, as shown in Fig. 4a. Notably, in this region, due to the strong Rashba spin-orbit coupling strength ${\alpha }_{R}$, the density of states for the inner Rashba band is strongly suppressed. Our estimation shows that the density of states of outer Rashba band is an order of magnitude larger than the inner one. This indicates that the transport is likely dominated by the outer band, which explains the observed single-band transport behaviour. Moreover, a previous study on BiTeBr^46^ with similar carrier densities has found that Coulomb impurities with a screening wavevector much smaller than the Fermi-wavevector difference between the two Rashba bands is the dominant scattering source. This further supports the independent-band transport picture and the dominance of the outer Rashba band in NRMT. This identification in combination with the high mobility also helps exclude another NRMT mechanism of quartic scaling^35^, suggesting that the LSK is left as the uniquely predominant candidate (Methods).

Moreover, adopting the above effective model and modelling skew scattering processes from the screened Coulomb impurities, we were able to estimate the LSK induced NRMT coefficient $\chi$ (details in Methods). The results are shown in Fig. 4b along with the experimental result. The agreement is excellent. The theoretical result reproduces not only the temperature dependence of $\chi$ but also its correct order of magnitude. For example, at 100 K, the theoretical estimation for Device#1 gives ${\chi }_{\perp }^{2\omega }\sim 3.48\times {10}^{-4}{{{\rm{A}}}}{{{{\rm{V}}}}}^{-2}{{{{\rm{T}}}}}^{-1}$, which is close to the experimental value of ${\chi }_{\perp }^{2\omega }\sim 3.68\times {10}^{-4}{{{\rm{A}}}}{{{{\rm{V}}}}}^{-2}{{{{\rm{T}}}}}^{-1}$. Such quantitative agreement offers strong support that LSK is the dominant mechanism underlying the observed NRMT response here.

To investigate the crossover from our observed LSK mechanism (${\sigma }_{{xx}}^{4}$ scaling) in high-mobility BiTeBr samples to the previously reported Zeeman-coupling mechanism (${\sigma }_{{xx}}^{2}$ scaling) in BiTeBr with lower mobilities^4^, and to identify whether mobility is a key parameter that determines the dominant origin of NRMT, we fabricated a series of devices (Device #1–#10) spanning a wide mobility range. We measured their NRMT signals and analysed the corresponding scaling behaviour. The basic characteristics of each BiTeBr device and the corresponding dominant mechanisms are summarised in Supplementary Table S3, and their transport properties and NRMT responses are presented in Supplementary Figs S11–S17. The variation of the dominant ${\sigma }_{{xx}}$-scaling with the low-temperature (2 K) device mobility is shown in Fig. 4c, which exhibits a clear crossover behaviour. Devices with relatively high mobilities (~ 2000 cm^2^V^–1^s^−1^ at 2 K), such as Devices #1, #4, and #5, show NRMT responses dominated by the ${\sigma }_{{xx}}^{4}$ term. In contrast, low-mobility devices (~ 420 cm^2^V^−1^s^−1^ at 2 K), such as Device #10, exhibit NRMT arising almost entirely from the ${\sigma }_{{xx}}^{2}$ term. This trend is consistent with previous BiTeBr work^4^, where devices with mobility ranging from 143 to 494 cm²V⁻¹s⁻¹ at 2 K shows NRMT dominated by the ${\sigma }_{{xx}}^{2}$ term. Devices with intermediate mobilities (Devices #7–#9, mobilities ~900 cm^2^V^−1^s^−1^ at 2 K) display mixed behaviour, with comparable contributions from both ${\sigma }_{{xx}}^{4}$ and ${\sigma }_{{xx}}^{2}$ terms. Device #6 with an intermediate but higher mobility (~1220 cm^2^V^−1^s^−1^ at 2 K), is dominated by the ${\sigma }_{{xx}}^{4}$ scaling but shows a non-negligible ${\sigma }_{{xx}}^{2}$ contribution at elevated temperatures, placing it near the crossover between purely LSK-dominated and mixed-mechanism regimes.

In conclusion, we have reported a distinct quartic scaling behaviour of NRMT response in high-mobility BiTeBr samples. Via systematic analysis, we reveal that such nonreciprocal transport originates from a LSK mechanism, in which the magnetic field enters via Lorentz force rather than Zeeman coupling and the quantum geometric character manifests in the skew scattering rate. The topological band structures on the n-doping side help boost the mobility of electrons which makes LSK gain dominance due to its high scaling power in relaxation time. Meanwhile, it should be noted that the LSK mechanism itself does not require spin-orbit coupling, so it may also be observed in materials with negligible spin-orbit coupling strength. Due to its quartic scaling behaviour, the LSK induced nonreciprocal transport should be greatly enhanced in materials with long electronic relaxation time and strong Berry curvatures on Fermi surface. This implies that topological materials, such as graphene superlattices and Weyl semimetals (where $\tau$ may reach 10 to 100 ps), could be good platforms to achieve large NRMT. Our work thus suggests a route to giant transport nonreciprocity in high-mobility materials with low dissipation and power consumption, which holds great promise for efficient nonlinear devices such as rectifiers and diodes.

## Methods

### Device fabrication

The BiTeBr crystals were grown using a BiBr_3_ flux method. Bi_2_Te_3_ and BiBr_3_ were mixed in a molar ratio of 1:9 and sealed in a quartz ampoule. The mixture was heated to 400 °C over 3 h and held for 10 h, followed by heating to 600 °C in 1.5 h and soaking at this temperature for one day. The melt was then crystallised using a modified Bridgman technique. To further improve the crystal quality and enhance mobility, the as-grown crystals were annealed with a Bi_2_Te_3_–BiBr_3_ mixture at 200 °C for three days. This post-annealing process helps reduce defects and compositional inhomogeneity, which are known to limit carrier mobility in the BiTeBr system.

The BiTeBr flakes were mechanically exfoliated on a SiO_2_/Si substrate inside nitrogen-filled glovebox with O_2_ and H_2_O level kept below 1 ppm. The crystalline axes of the exfoliated BiTeBr flakes were determined using the second harmonic generation (SHG) method. To prevent degradation, a PMMA polymer layer was coated on BiTeBr flakes inside glove box before SHG measurement. To enhance electrode contact, the stencil mask method was employed to deposit metal contacts (Cr/Au, 2/50 nm) on the samples. To standardise the shape of the device and avoid geometrical complications in the current path, reactive ion etching with SF6 and CHF3 gases were used. After all fabrication processes, a PMMA polymer layer was applied once again to prevent degradation before measurement.

### Nonreciprocal magneto-transport measurements

The nonreciprocal magneto-transport measurements were conducted in a Quantum Design PPMS cryostat using a Horizontal Rotator, allowing the BiTeBr devices to be rotated through a full 360° in the presence of an applied magnetic field. A harmonic current was applied to the BiTeBr devices during the measurement, and second harmonic voltage drops in both longitudinal and transverse directions were recorded using a standard lock-in technique (Zurich MFLI). An in-plane magnetic field ranging from 1 to 9 T was applied throughout the measurements, with the device being rotated by the Horizontal Rotator around its *x*-axis at an angle *θ* relative to the magnetic field direction. The phases of the first- and second- harmonic signals were confirmed to be approximately 0° and 90°, respectively, during all electrical measurements.

### Symmetry analysis of angular dependence of NRMT

For a setup with in-plane electric field ${{{\bf{E}}}}=({E}_{x},{E}_{y},0)$ and in-plane magnetic field ${{{\bf{B}}}}=({B}_{x},{B}_{y},0)$, the NRMT is described by ${j}_{a}={\chi }_{{abcd}}{E}_{b}{E}_{c}{B}_{d}$, in which ${\chi }_{{abcd}}$ is the nonlinear planar conductivity. Here indices $a,b,c,d$ denote the Cartesian coordinates $\{x,y\}$. Imposing the ${C}_{3v}$ symmetry on the fourth-rank axial tensor ${\chi }_{{abcd}}$ reduces the allowed in-plane components to ${\chi }_{{xxxy}}$, ${\chi }_{{xyxx}}$, ${\chi }_{{yxxx}}$, ${\chi }_{{xyyy}}$, ${\chi }_{{yxyy}}$, ${\chi }_{{yyxy}}$, ${\chi }_{{yyyx}}$, ${\chi }_{{xxyx}}$, which satisfy

$${\chi }_{{yxxx}}={\chi }_{{yyyx}}+{\chi }_{{yyxy}}+{\chi }_{{yxyy}},$$

$${\chi }_{{xyyy}}=-{\chi }_{{yxxx}},$$

$${\chi }_{{xyxx}}=-{\chi }_{{yxyy}},$$

$${\chi }_{{xxyx}}=-{\chi }_{{yyxy}},$$

$${\chi }_{{xxxy}}=-{\chi }_{{yyyx}}.$$

Using these symmetry relations, the second order current density can be expressed as

$${{{{\bf{j}}}}}^{2{{{\rm{\omega }}}}}=\left(\begin{array}{c}-\left({\chi }_{1}+{\chi }_{2}\right){E}_{x}{E}_{y}{B}_{x}+{\chi }_{1}{E}_{x}^{2}{B}_{y}-{\chi }_{2}{E}_{y}^{2}{B}_{y}\\ {\chi }_{2}{E}_{x}^{2}{B}_{x}-{\chi }_{1}{E}_{y}^{2}{B}_{x}+\left({\chi }_{1}+{\chi }_{2}\right){E}_{x}{E}_{y}{B}_{y}\\ 0\end{array}\right),$$

where ${\chi }_{1}\equiv {\chi }_{{xxxy}}$ and ${\chi }_{2}\equiv {\chi }_{{yxxx}}$ are short notations. The electric field induced by ${{{{\bf{j}}}}}^{2{{{\rm{\omega }}}}}$ is obtained by the relation ${{{{\bf{E}}}}}^{2\omega }=\rho {{{{\bf{j}}}}}^{2{{{\rm{\omega }}}}}$. The ${C}_{3v}$ symmetry enforces an isotropic *B*-independent in-plane resistivity $\rho={\rho }_{0}$ up to the linear order of *B* field, which is the concerned order here. Consequently, the second-order electric field components maintain the same directionality as the current components:

$${{{{\bf{E}}}}}^{2\omega }={\rho }_{0}\left(\begin{array}{c}-\left({\chi }_{1}+{\chi }_{2}\right){E}_{x}{E}_{y}{B}_{x}+{\chi }_{1}{E}_{x}^{2}{B}_{y}-{\chi }_{2}{E}_{y}^{2}{B}_{y}\\ {\chi }_{2}{E}_{x}^{2}{B}_{x}-{\chi }_{1}{E}_{y}^{2}{B}_{x}+\left({\chi }_{1}+{\chi }_{2}\right){E}_{x}{E}_{y}{B}_{y}\end{array}\right).$$

In the experimental configuration, we applied an in-plane driving current density ${{{\bf{j}}}}=j\left(\cos \varphi,\sin \varphi \right)$, which generates a *B*-independent longitudinal driving field ${{{\bf{E}}}}=j{\rho }_{0}\left(\cos \varphi,\sin \varphi \right)$ up to the linear order of *B* field. Therefore, the induced second-order electric field $\propto {E}^{2}B$ takes the form of

$${{{{\bf{E}}}}}^{2\omega }={E}^{2}B{\rho }_{0}\left(\begin{array}{c}-\left({\chi }_{1}+{\chi }_{2}\right)\cos \varphi \sin \varphi \cos \theta+{\chi }_{1}{\cos }^{2}\varphi \sin \theta -{\chi }_{2}{\sin }^{2}\varphi \sin \theta \\ {\chi }_{2}{\cos }^{2}\varphi \cos \theta -{\chi }_{1}{\sin }^{2}\varphi \cos \theta+\left({\chi }_{1}+{\chi }_{2}\right)\cos \varphi \sin \varphi \sin \theta \end{array}\right),$$

where $\theta$ is the orientation of in-plane *B* field. The transverse component ${E}_{\perp }^{2\omega }$ is given by ${E}_{\perp }^{2\omega }=\left(-\sin \varphi,\cos \varphi \right)\cdot {{{{\bf{E}}}}}^{2\omega }$, and the longitudinal component is ${E}_{{||}}^{2\omega }=\left(\cos \varphi,\sin \varphi \right)\cdot {{{{\bf{E}}}}}^{2\omega }$. One thus obtains (here we use the notation ${E}_{{||}}$ for *E*, which is adopted in the main text description of experiments)

$$\frac{{E}_{\perp }^{2\omega }}{{({E}_{{||}})}^{2}B}={\rho }_{0}{\chi }_{2}\cos \left(\theta -\varphi \right),\, \frac{{E}_{{||}}^{2\omega }}{{({E}_{{||}})}^{2}B}={\rho }_{0}{\chi }_{1}\sin \left(\theta -\varphi \right).$$

The ${C}_{3v}$ symmetry enforces ${\rho }_{0}=1/\sigma$, where $\sigma$ is the zero-field longitudinal conductivity. As such,

$$\sigma \frac{{E}_{\perp }^{2\omega }}{{({E}_{{||}})}^{2}B}={\chi }_{2}\cos \left(\theta -\varphi \right),\, \sigma \frac{{E}_{{||}}^{2\omega }}{{({E}_{{||}})}^{2}B}={\chi }_{1}\sin \left(\theta -\varphi \right).$$

Identifying $\hat{{{{\bf{P}}}}}$ as the polar axis (along z), one obtains

$${\chi }_{\perp }\equiv \sigma \frac{{E}_{\perp }^{2\omega }}{{({E}_{{||}})}^{2}B}\propto {\chi }_{2}{{{\bf{I}}}}\cdot {{{\bf{B}}}},\, {\chi }_{{||}}\equiv \sigma \frac{{E}_{{||}}^{2\omega }}{{({E}_{{||}})}^{2}B}\propto {\chi }_{1}\left({{{\bf{I}}}}\times {{{\bf{B}}}}\right)\cdot \hat{{{{\bf{P}}}}},$$

which is precisely the outer product from $\sim \left({{{\bf{I}}}}\times {{{\bf{B}}}}\right)\cdot \hat{{{{\bf{P}}}}}$ and inner product form $\sim {{{\bf{I}}}}\cdot {{{\bf{B}}}}$ mentioned in the main text.

### Dominance of LSK in the quartic scaling behaviour of NRMT

A recent theory on the second order electrical nonlinear transport $\propto {E}^{2}$ in magnetic systems predicted quartic scaling from the composition of two skew scattering processes (SKSK)^35^. This contribution is also possible here as the nonmagnetic material can be viewed as being “magnetised” by the applied magnetic field. We now inspect this possibility in our devices. From the perspective of scaling law, a more detailed scaling-law analysis^50^ shows that ${\chi }^{{LSK}}\sim {\tau }^{4}/{\tau }^{{sk}}$ and ${\chi }^{{SKSK}}\sim {\tau }^{4}/{\left({\tau }^{{sk}}\right)}^{2}$, where ${\tau }^{{sk}}$ is a time scale related to skew scattering and is much larger than $\tau$^39,54,55^. Since $1/{\tau }^{{sk}}\sim {n}_{i}$, with ${n}_{i}$ being the impurity concentration, $\frac{{\chi }^{{{{\rm{LSK}}}}}}{{\chi }^{{{{\rm{SKSK}}}}}}$ is insensitive to temperature, and ${\chi }^{{LSK}}$ should be much larger than ${\chi }^{{SKSK}}$ in materials with smaller ${n}_{i}$, i.e., high low-temperature mobility. The ratio between LSK and the Zeeman corrected SKSK contribution is approximately given by $\frac{{\chi }^{{{{\rm{LSK}}}}}}{{\chi }^{{{{\rm{SKSK}}}}}}\sim \sigma {\omega }_{c}\tau /\frac{{c}_{1}\Delta }{{{{\hslash }}{{{\rm{v}}}}}_{{{{\rm{F}}}}}{k}_{F}}\sigma \frac{{g\mu }_{B}B}{\Delta }\sim \frac{2{{{\hslash }}{{{\rm{v}}}}}_{{{{\rm{F}}}}}{k}_{F}\tau }{{\hslash }g{c}_{1}}$, where ${\omega }_{c}$ is the cyclotron frequency, ${\mu }_{B}$ is the Bohr magneton, $g$ is the *g*-factor, $\Delta$ is the vertical interband separation, and ${c}_{1}=\tau /{\tau }^{{sk}}$ is much less than unity^39,54–56^. ${{\hslash }{{{\rm{v}}}}}_{{{{\rm{F}}}}}{k}_{F}$ is a characteristic energy scale related to electronic motion on the Fermi surface^46^. The estimated relaxation times according to the measured mobility at 40 K are large, reaching ${{{\rm{\tau }}}}\simeq 0.15{{{\rm{ps}}}}$ for Device#1 and Device#4 (Supplementary Table S2). In addition, the ratio $\frac{{\chi }^{{{{\rm{LSK}}}}}}{{\chi }^{{{{\rm{SKSK}}}}}}$ is further enhanced by the large Fermi velocity and Fermi wavevector of the outer Rashba band (${{\hslash }{{{\rm{v}}}}}_{{{{\rm{F}}}}}{k}_{F}\simeq 147$ meV in Device#1 and ${{\hslash }{{{\rm{v}}}}}_{{{{\rm{F}}}}}{k}_{F}\simeq 212$ meV in Device#4). Therefore, assuming a considerable value of ${c}_{1}=0.1$ and the *g*-factor $g=60$^4^, the LSK is more than one order of magnitude larger than the SKSK in our devices, giving the overwhelmingly dominant quartic-scaling contribution.

### First-principles calculations

The first-principles calculations are based on the density functional theory (DFT), using the projector-augmented wave method^57^ as implemented in the Vienna ab-initio simulation package (VASP)^58,59^. The Perdew-Burke-Ernzerhof (PBE) functional was utilised to capture exchange-correlation effects^60^. The cutoff energy is 400 eV. The lattice structures are relaxed with an energy convergence criterion of 10^−8^ eV and a force convergence criterion of 0.01 eV/Å. The Brillouin zone is sampled by a Γ-centred Monkhorst-Pack k-point mesh^61^ with size 15 × 15 × 7 for self-consistent calculations. Spin-orbit coupling is included in BiTeBr calculations. Wannier tight-binding model with p orbitals of Bi, Te and Br atoms was constructed by using the Wannier90 package^62–64^.

### Skew scattering in three-dimensional Rashba bands

The BiTeBr has ${C}_{3v}$ point group symmetry generated by a three-fold rotation ${{{{\mathcal{C}}}}}_{3z}$ and mirror symmetry ${{{{\mathcal{M}}}}}_{x}$. Its low-energy Hamiltonian can be captured by a three-dimensional Rashba model

$${{{\mathcal{H}}}}=t({k}_{x}^{2}+{k}_{y}^{2})+{t}_{z}{k}_{z}^{2}+{\alpha }_{R}({k}_{y}{\sigma }_{x}-{k}_{x}{\sigma }_{y})+({k}_{+}^{3}+{k}_{-}^{3})\left(\frac{\lambda {\sigma }_{z}}{2}+\frac{{\lambda }_{0}{k}_{z}}{2}\right),$$

measured from A point. Here, ${k}_{\pm }={k}_{x}\pm i{k}_{y}$, and $\sigma$ denotes Pauli matrix. The third term represents Rashba spin-orbit coupling, $\lambda$ term added hexagonal warping of the Fermi surface, and ${\lambda }_{0}$ term deforms the hexagonal warping to a trigonal warping at nonzero ${k}_{z}$ as required by ${{{{\mathcal{C}}}}}_{3z}$. In our calculations, we take model parameters as *t* = 25eVÅ^2^, *t*_*z*_ = 5eVÅ^2^, *α*_*R*_ = 2.0eVÅ, *λ* = 50eVÅ^3^, *λ*_0_ = 20eVÅ^4^, which can reproduce the band structures from first-principles calculations.

The energy dispersion of this model is

$$\epsilon (k)\approx 	 \,{t}_{z}{k}_{z}^{2}+t({k}_{x}^{2}+{k}_{y}^{2})+\eta \sqrt{{\lambda }^{2}{({k}_{x}^{3}-{k}_{x}{k}_{y}^{2})}^{2}+{\alpha }_{R}({k}_{x}^{2}+{k}_{y}^{2})} \\ 	+{\lambda }_{0}\left({k}_{x}^{3}-{k}_{x}{k}_{y}^{2}\right){k}_{z},$$

where $\eta=\pm 1$, and the corresponding eigenstates are

$$|{u}_{{{{\boldsymbol{k}}}}}^{+}{{\rangle }}=\left(\begin{array}{c}\cos (\theta /2)\\ -i\sin (\theta /2){e}^{i\phi }\end{array}\right),|{u}_{{{{\boldsymbol{k}}}}}^{-}{{\rangle }}=\left(\begin{array}{c}-i\sin (\theta /2)\\ \cos (\theta /2){e}^{i\phi }\end{array}\right).$$

Here, ${k}_{\perp }=\scriptstyle\sqrt{{k}_{x}^{2}+{k}_{y}^{2}}$, $\tan \phi=\frac{{k}_{y}}{{k}_{x}}$, and $\cos \theta=\frac{\lambda {k}_{\perp }^{2}\cos 3\phi }{\sqrt{{\alpha }_{R}^{2}+{(\lambda {k}_{\perp }^{2}\cos 3\phi )}^{2}}}$. The leading term of the Bloch state overlap is

$$\langle {u}_{{{{\boldsymbol{k}}}}}^{+}|{u}_{{{{{\boldsymbol{k}}}}}^{{{{\prime} }}}}^{+}{{\rangle }}=	 \langle {u}_{{{{{\boldsymbol{k}}}}}^{{{{\prime} }}}}^{-}|{u}_{{{{\boldsymbol{k}}}}}^{-}{{\rangle }}\approx \frac{1}{2}(1+{e}^{i({\phi }^{{\prime} }-\phi )}) \\ 	+\frac{\lambda }{4{\alpha }_{R}}({k}_{\perp }^{2}\cos 3\phi+{k}_{\perp }^{{\prime} 2}\cos 3{\phi }^{{\prime} })(1-{e}^{i({\phi }^{{\prime} }-\phi )})+{{{\mathscr{O}}}}({\lambda }^{2}),$$

Consider the screened Coulomb scattering (Gaussian-natural units) ${{{{\rm{V}}}}}_{{{{\boldsymbol{kk}}}}{{^{\prime} }}}=\frac{4\pi {e}^{2}\alpha }{{({{{\boldsymbol{k}}}}-{{{{\boldsymbol{k}}}}}^{{{{\prime} }}})}^{2}+{q}_{{TF}}^{2}}\langle {u}_{{{{\boldsymbol{k}}}}}| {u}_{{{{{\boldsymbol{k}}}}}^{{\prime} }}\rangle$, where ${\alpha }^{-1}\approx 16$ is the relative dielectric constant of BiTeBr, we get the lowest Born scattering rate

$${\omega }_{{{{\boldsymbol{k}}}},{{{\boldsymbol{k}}}}^{\prime} }^{\pm (2)} 	=\frac{2\pi }{{\hslash }}\langle {{{{\rm{V}}}}}_{{{{\boldsymbol{kk}}}}^{\prime} }{{{{\rm{V}}}}}_{{{{\boldsymbol{k}}}}^{\prime} {{{\boldsymbol{k}}}}}{{{\rangle }}}_{{dis}}\delta ({\epsilon }_{{{{\boldsymbol{k}}}}}-{\epsilon }_{{{{{\boldsymbol{k}}}}}^{{\prime} }}) \\ 	=\frac{2\pi }{{\hslash }}{n}_{i}{\left(\frac{4\pi {e}^{2}\alpha }{{({{{\boldsymbol{k}}}}-{{{{\boldsymbol{k}}}}}^{{\prime} })}^{2}+{q}_{{TF}}^{2}}\right)}^{2}\left(1+\cos \left({\phi }^{{\prime} }-\phi \right)\right)\delta ({\epsilon }_{{{{\boldsymbol{k}}}}}-{\epsilon }_{{{{{\boldsymbol{k}}}}}^{{\prime} }}){{{\mathscr{+}}}}{{{\mathscr{O}}}}\left(\lambda \right),$$

and the antisymmetric scattering rate responsible for skew scattering

$$	{\omega }_{{{{\boldsymbol{k}}}},{{{{\boldsymbol{k}}}}}^{{\prime} }}^{\pm \left(3a\right)}=\frac{4{\pi }^{2}}{{\hslash }}{\sum}_{{{{{\boldsymbol{k}}}}}^{{{{\prime} }}{{{\prime} }}}}{\mathfrak{I}} \langle {{{{\rm{V}}}}}_{{{{\boldsymbol{k}}}}{{{{\boldsymbol{k}}}}}^{{{{\prime} }}{{{\prime} }}}}{{{{\rm{V}}}}}_{{{{{\boldsymbol{k}}}}}^{{{{\prime} }}{{{\prime} }}}{{{{\boldsymbol{k}}}}}^{{{{\prime} }}}}{{{{{\rm{V}}}}}_{{{{{\boldsymbol{k}}}}}^{{{{\prime} }}}{{{\boldsymbol{k}}}}} \rangle}_{{dis}}\delta \left({\epsilon }_{{{{\boldsymbol{k}}}}}-{\epsilon }_{{{{{\boldsymbol{k}}}}}^{{{{\prime} }}{{{\prime} }}}}\right)\delta \left({\epsilon }_{{{{\boldsymbol{k}}}}}-{\epsilon }_{{{{{\boldsymbol{k}}}}}^{{{{\prime} }}}}\right) \\ 	=\mp \frac{\lambda }{2\pi {\hslash }{\alpha }_{R}}\delta ({\epsilon }_{{{{\boldsymbol{k}}}}}-{\epsilon }_{{{{{\boldsymbol{k}}}}}^{{{{\prime} }}}})\int d{{{{\boldsymbol{k}}}}}^{{\prime} {\prime}} [\sin \left({\phi }^{\prime} - \phi \right){k}_{\perp }^{{\prime} {\prime} 2} \cos 3{\phi }^{{\prime} {\prime} }+\sin \left({\phi }^{{\prime} {\prime} }-{\phi }^{{\prime} }\right){k}_{\perp }^{2}\cos 3\phi \\ 	+\sin ({\phi -\phi }^{{\prime} {\prime} }){k}_{\perp }^{{\prime} 2}cos 3{\phi }^{{\prime} }]\frac{4\pi {e}^{2}\alpha }{{({{{\boldsymbol{k}}}}-{{{\boldsymbol{k}}}}^{\prime} )}^{2}+{q}_{{TF}}^{2}}\frac{4\pi {e}^{2}\alpha }{{({{{\boldsymbol{k}}}}^{\prime} -{{{\boldsymbol{k}}}}^{\prime\prime} )}^{2}+{q}_{{TF}}^{2}}\frac{4\pi {e}^{2}\alpha }{{({{{\boldsymbol{k}}}}^{\prime\prime} -{{{\boldsymbol{k}}}})}^{2}+{q}_{{TF}}^{2}}\delta ({\epsilon }_{{{{\boldsymbol{k}}}}}-{\epsilon }_{{{{{\boldsymbol{k}}}}}^{{{{\prime} }}{{{\prime} }}}})$$

for the outer ($-$) and inner ($+$) Rashba bands. Substituting the above equations into the LSK distribution function [Eq. (1) of the main text] we can get the expressions for the LSK nonlinear current. Given the complexity of high-dimensional numerical integrals, we adopt semiquantitative estimation to get meaningful expressions for the LSK nonlinear conductivity. The characteristic Coulomb potential is approximated as $\frac{4\pi {e}^{2}\alpha }{{k}_{F}^{2}}$, then the LSK nonlinear conductivity at the second harmonic can be simplified into

$${\chi }^{2{{{\rm{\omega }}}},{{{\rm{LSK}}}}}\approx \frac{{2}^{11}{\pi }^{5}{\tau }^{4}{e}^{10}{\alpha }^{3}{n}_{i}{t}^{2}\lambda {\lambda }_{0}}{{{\hslash }}^{6}{\alpha }_{R}}\frac{{D}_{F}^{3}}{{k}_{F}}.$$

Here, ${D}_{F}$ is the density of states at the Fermi level. The factor $\frac{{D}_{F}^{3}}{{k}_{F}}$ for the outer Rashba band is 77 times of that for the inner band in Device#1, which is consistent with the conclusion drawn from qualitative model analysis and out-of-plane Hall measurement, supporting also the validity of our estimation procedure. Assuming that the charged impurity density is approximately the same as the carrier density, we then get the theoretical results shown in Fig. 4b.

## Supplementary information

Supplementary Information

Transparent Peer Review file

## Source data

Source Data

## Data Availability

The data that supports the findings of this study are provided as source data files with this paper. Source Data file has been deposited in Zenodo under accession code (10.5281/zenodo.18952454). All data is available from the corresponding authors upon request. Source data are provided with this paper.
