# Supplementary material for: Lorentz skew scattering nonreciprocal magneto-transport
Source: Nat Commun. 2026 May 8;17:6221. doi: 10.1038/s41467-026-71789-y (PMC13369428; doi:10.1038/s41467-026-71789-y)
Supplement: Supplementary file 1 — Supplementary Information [file 41467_2026_71789_MOESM1_ESM.pdf]

Supplementary Information

Lorentz Skew Scattering Nonreciprocal Magneto-Transport

Xiu Fang Lu[1,11], Xue-Jin Zhang[2,11], Naizhou Wang[3*], Jin Cao[2], Dan Zhao[4], Hui Wang[5], Tao Wu[4], Xianhui Chen[4], Shen Lai[2], Shuigang Xu[3], Cong Xiao[6*], Shengyuan A. Yang[7*], and Weibo Gao[5,8,9,10*]

[1]Hangzhou International Innovation Institute, Beihang University, Hangzhou, China.

[2]Institute of Applied Physics and Materials Engineering, Faculty of Science and Technology, University of Macau, Macau SAR, China.

[3]Department of Physics, School of Science, Westlake University, Hangzhou 310024, China.

[4]Department of Physics and Hefei National Laboratory for Physical Science at Microscale, University of Science and Technology of China, Hefei, Anhui 230026, China.

[5]Division of Physics and Applied Physics, School of Physical and Mathematical Sciences, Nanyang Technological University, Singapore 637371, Singapore.

[6]Interdisciplinary Center for Theoretical Physics and Information Sciences (ICTPIS), Fudan University, Shanghai 200433, China

[7]Research Laboratory for Quantum Materials, Department of Applied Physics, The Hong Kong Polytechnic University, Hong Kong, China

[8]School of Electrical and Electronic Engineering, Nanyang Technological University, Singapore, Singapore

[9]Centre for Quantum Technologies, Nanyang Technological University, Singapore, Singapore

[10]National Centre for Advanced Integrated Photonics (NCAIP) Singapore, Nanyang Technological University, Singapore, Singapore

[11]These authors contributed equally: Xiu Fang Lu, Xue-Jin Zhang

E-mail: wbgao@ntu.edu.sg, shengyuan.yang@polyu.edu.hk, congxiao@fudan.edu.cn, wangnaizhou@westlake.edu.cn

This file includes:

S1. Basic device characteristics of BiTeBr Device #1

S2. Fundamental Nonreciprocal magneto-transport (NRMT) response of BiTeBr device

S3. Scaling analysis of NRMT response and quantitative evaluation of the σ_{xx}^{n} (n = 0 to 4) contribution

S4. Additional data for BiTeBr Devices #4 to #10

S5. First-principles band structures calculations of BiTeBr

S1. Basic device characteristics of BiTeBr Device #1

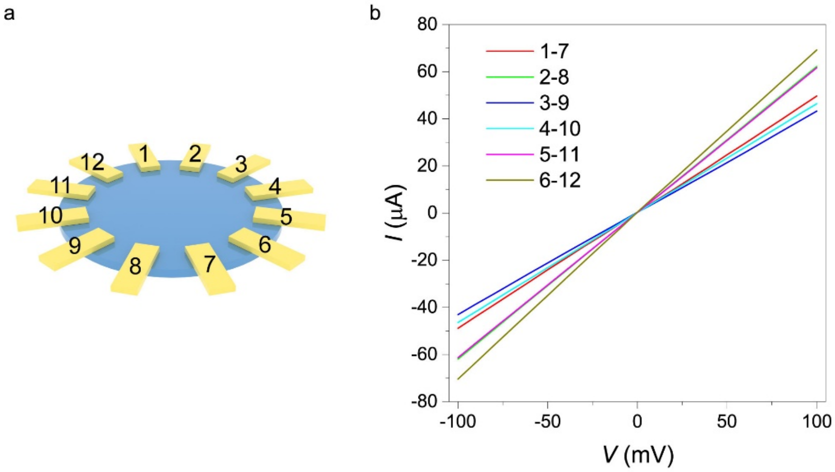

Supplementary Figure S1 | Two-terminal contact characteristics of disc-shape BiTeBr Device #1. a, Schematic illustration of Device #1 with labelled electrode numbers. **b**, Two-terminal *I*-*V* curves for all electrodes.

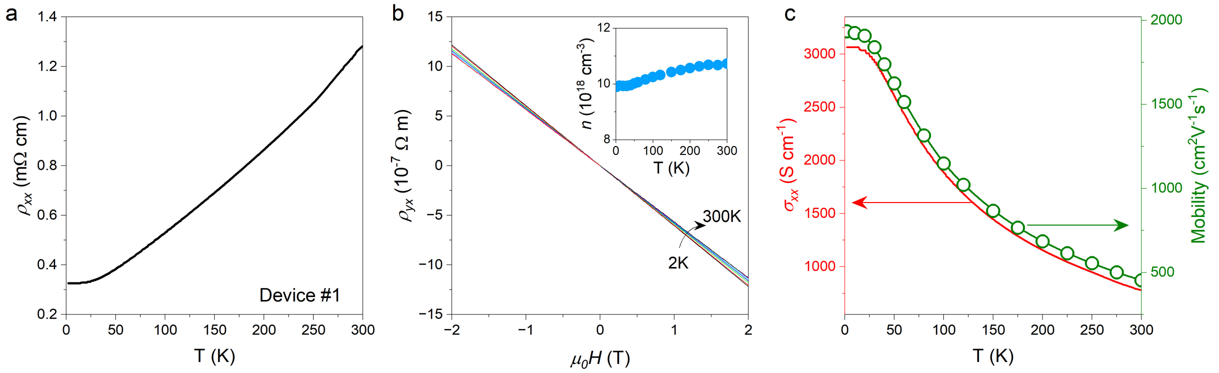

Supplementary Figure S2 | The resistivity, carrier density, conductivity, and mobility of BiTeBr Device #1. **a**, Temperature dependence of reisistivity. **b**, Hall resistivity ρ_{yx} measured at temperature ranging from 2 to 300 K. Insert is the carrier density obtained from Hall resistivity. **c**, longitudinal conductivity (left) and mobility (right) of BiTeBr Device #1 at temperature ranging from 2 to 300 K.

S2. Fundamental Nonreciprocal magneto-transport (NRMT) response of BiTeBr device

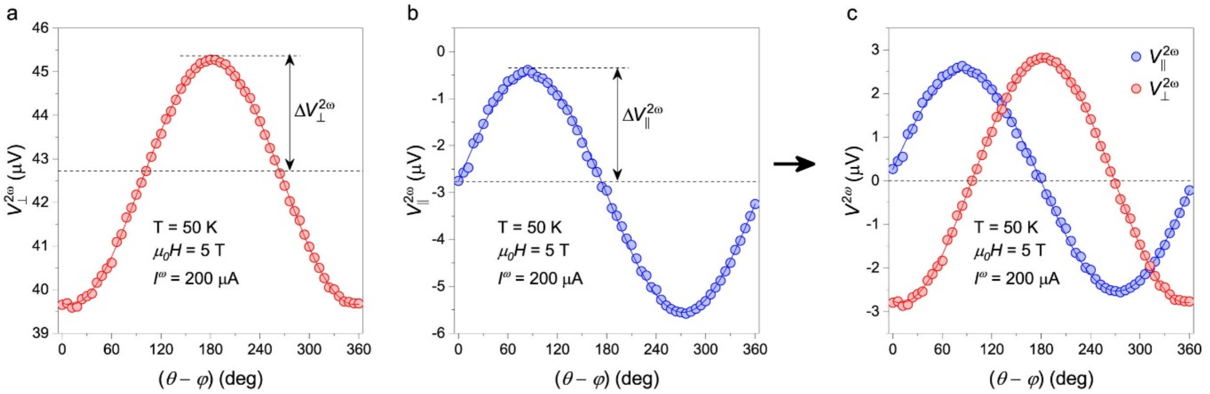

Supplementary Figure S3 | Background subtraction of sine and cosine curves for $V_{\parallel}^{2\omega}$ and $V_{\perp}^{2\omega}$ arising from the B-independent second-harmonic response. a and **b**, $V_{\perp}^{2\omega}$ and $V_{\parallel}^{2\omega}$ as a function of angle $(\theta - \varphi)$, respectively. **c**, $V_{\perp}^{2\omega}$ and $V_{\parallel}^{2\omega}$ are shown in a combined plot after subtracting the background contributions of sine and cosine curves due to the B-independent second-harmonic response. This method isolates the data driven purely by the in-plane magnetic field for clarity.

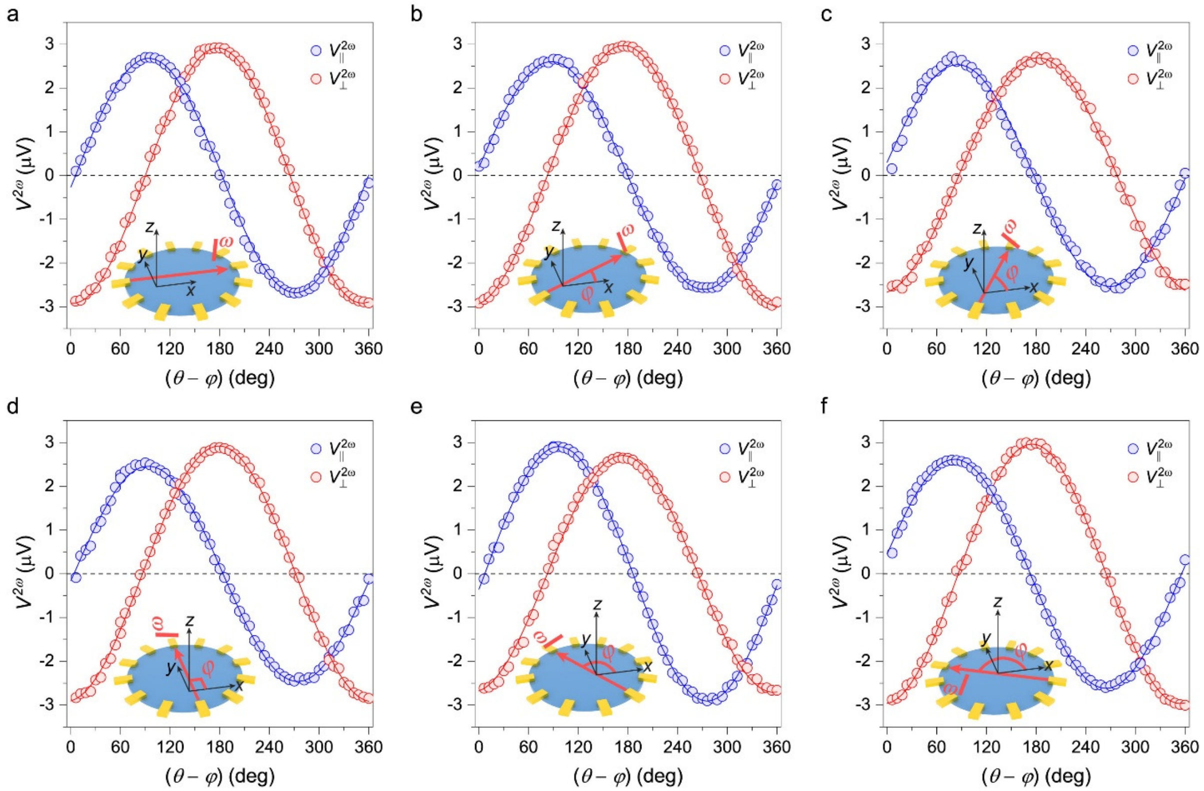

Supplementary Figure S4 | The NRMT responses, $V_{\parallel}^{2\omega}$ and $V_{\perp}^{2\omega}$, with I^{ω} applied along 6 different directions at 30° intervals in a disc-shape BiTeBr Device #1. a-f, Dependence of $V_{\parallel}^{2\omega}$ and $V_{\perp}^{2\omega}$ on angle ($\theta - \varphi$) for measurements with I^{ω} applied along 6 distinct directions at 30° intervals. Measurements were conducted with $I^{\omega} = 200$ μA and a 5 T in-plane magnetic field at 50K. Inserts schematically illustrate the directions of the applied I^{ω}.

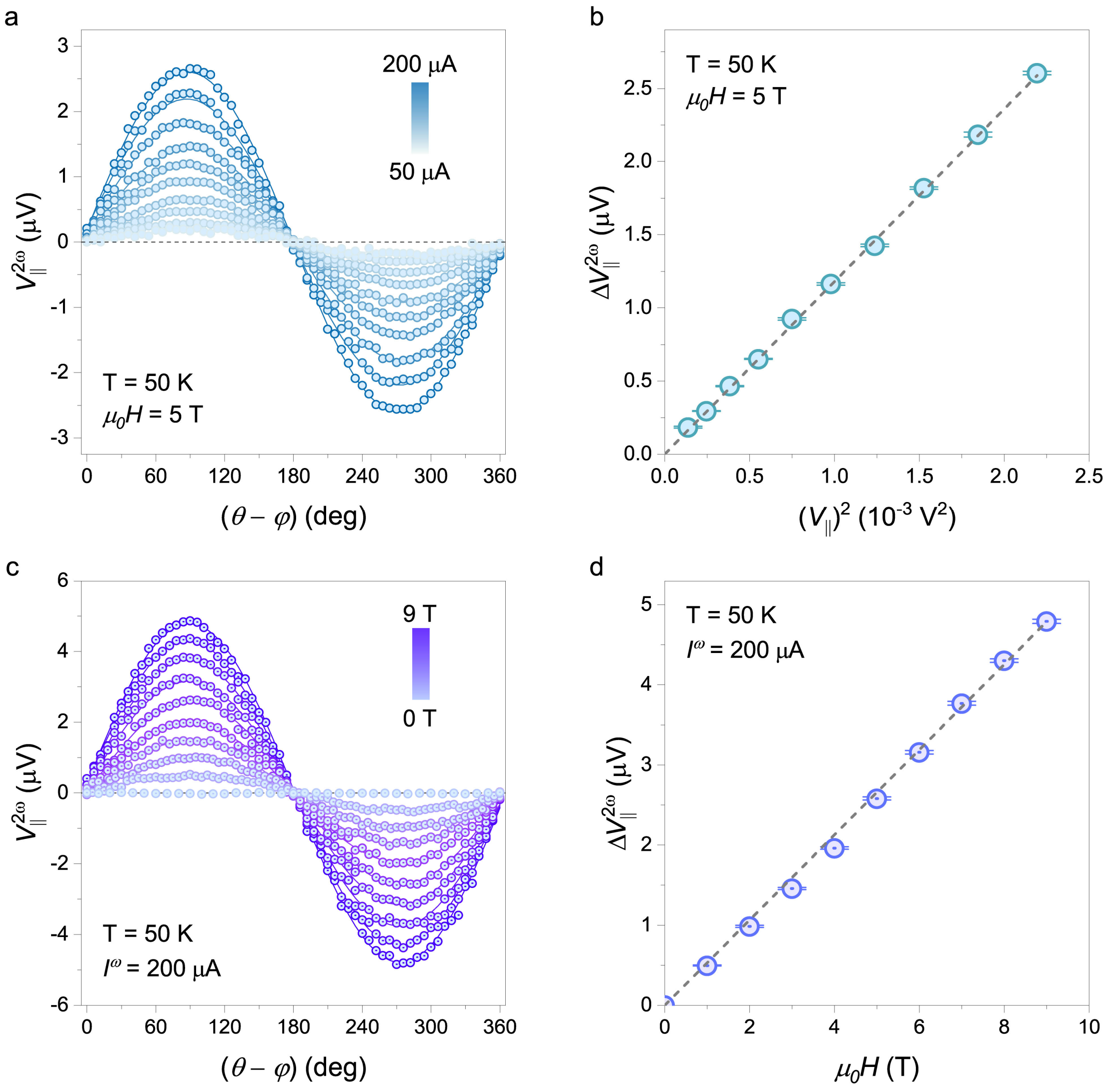

Supplementary Figure S5 | Nonreciprocal magneto-transport in the longitudinal direction under varying electric and magnetic field. **a**, **b**, Nonlinear longitudinal response $V_{||}^{2\omega}$ as a function of angle $(\theta - \varphi)$ measured with driving current I^{ω} from 50 to 200 μA (**a**), and the corresponding sinusoidal-fit amplitude $\Delta V_{||}^{2\omega}$ as a function of $(V_{||})^2$ (**b**). **c**, **d**, $V_{||}^{2\omega}$ as a function of angle $(\theta - \varphi)$ measured under in-plane magnetic field $\mu_0 H$ from 1 to 9 T (**c**), and the corresponding sinusoidal-fit amplitude $\Delta V_{||}^{2\omega}$ as a function of $\mu_0 H$ (**d**). Error bars in (**b**) and (**d**) represent the standard errors from the sinusoidal fits.

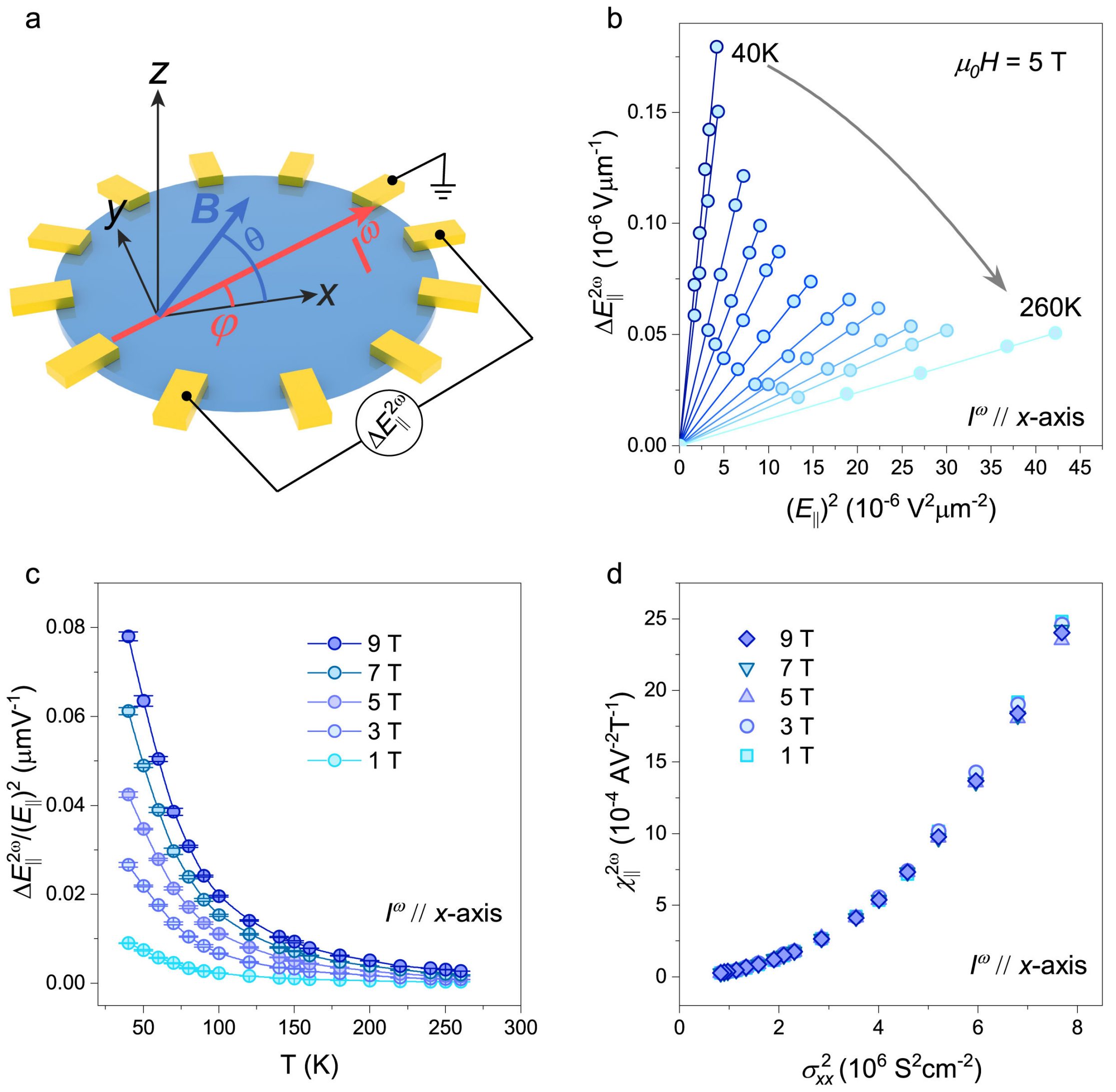

Supplementary Figure S6 | Temperature dependence of the longitudinal nonreciprocal magneto-transport in BiTeBr. a, Measurement configuration of the longitudinal nonreciprocal magneto-transport. **b,** $\Delta E_{\parallel}^{2\omega}$ depends linearly on $(E_{\parallel})^2$ from 40 to 260 K, measured under 5 T in-plane magnetic field with I^{ω} applied along *x*-axis. The slope of $\Delta E_{\parallel}^{2\omega}/(E_{\parallel})^2$ decreases with increasing temperature. **c**, $\Delta E_{\parallel}^{2\omega}/(E_{\parallel})^2$ as a function of temperature, measured under magnetic fields from 1 to 9 T. Error bars represent the standard errors of $\Delta E_{\parallel}^{2\omega}/(E_{\parallel})^2$ obtained from linear fits to $\Delta E_{\parallel}^{2\omega}$ - $(E_{\parallel})^2$ **d**, The longitudinal NRMT coefficient $\chi_{\parallel}^{2\omega}$ scaled as σ_{xx}^2. $\chi_{\parallel}^{2\omega}$ does NOT exhibit a linear dependence on σ_{xx}^2; instead, a higher order term in σ appears to contribute to the value of $\chi_{\parallel}^{2\omega}$.

S3. Scaling analysis of NRMT response and quantitative evaluation of the σ_{xx}^{n} (n = 0 to 4) contribution

To quantitative evaluate the contributions from each σ_{xx}^{n} (n = 0 to 4) term, we perform a systematic scaling analysis to Device #1 using alternative approachs, as described below.

Firstly, we plot the logarithmic plots of $\chi_{\perp}^{2\omega}$ and $\chi_{\parallel}^{2\omega}$ versus σ_{xx}, as shown in Fig. S7. The power extract from logarithmic plot are 3.93 and 4.01 for $\chi_{\perp}^{2\omega}$ and $\chi_{\parallel}^{2\omega}$, respectively, which consistent with the results that $\chi_{\perp}^{2\omega}$ and $\chi_{\parallel}^{2\omega}$ linear scale with σ_{xx}^{4}. Therefore, the σ_{xx}^{4} term is the dominant contribution for NRMT obseved in BiTeBr Device #1.

In addition, we plot $\chi_{\perp}^{2\omega}$ and $\chi_{\parallel}^{2\omega}$ of Device #1 as functions of σ_{xx}^{n} (n = 1 to 4). As the scaling order increases from first to fourth, $\chi_{\perp}^{2\omega}$ and $\chi_{\parallel}^{2\omega}$ exhibit progressively improved linearity, as shown in Fig. S8. Taken together these results consistently demonstrate that the NRMT response in Device #1 is dominated by the σ_{xx}^{4} scaling behavior, supporting the LSK mechanism as the primary origin.

To further assess the possible contribution of the Zeeman coupling (σ_{xx}^{2} term) to the NRMT in the Device #1, and to quantitatively disentangle the contribution from each σ_{xx}^{n} term (n = 0 to 4), we performed polynomial fits of $\chi_{\perp}^{2\omega}$ and $\chi_{\parallel}^{2\omega}$ using the following polynomial:

$$\chi^{2\omega} = A_4\sigma_{xx}^{4} + A_3\sigma_{xx}^{3} + A_2\sigma_{xx}^{2} + A_1\sigma_{xx}^{1} + A_0\sigma_{xx}^{0} \qquad (1)$$

where A_n represent the coefficient associated with the σ_{xx}^{n} contribution. Direct fourth-order polynomial (Poly4) fitting leads to an underdetermined system with many variables, resulting in a family of possible solutions. However, since a clear quartic scaling behavior ($\chi^{2\omega} \propto \sigma_{xx}^{4}$) is observed, we can extract A_4 and A_0 directly from the linear fit of $\chi^{2\omega}$ versus σ_{xx}^{4}, where the slope corresponds to A_4 and the intercept corresponds to A_0 (Fig. S8d, h). For $\chi_{\perp}^{2\omega}$, we obtain A_4 ~ 0.33 × 10^{-16} $AV^{-2}T^{-1}\cdot S^{-4}cm^{4}$, A_0 ~ 0.017 × 10^{-4} $AV^{-2}T^{-1}$. Since $\chi^{2\omega} = \sigma\frac{\Delta E^{2\omega}}{(E_{\parallel})^2 B}$, the term $\frac{\Delta E^{2\omega}}{(E_{\parallel})^2 B}$ can be written as: $\frac{\Delta E^{2\omega}}{(E_{\parallel})^2 B} = A_4\sigma_{xx}^{3} + A_3\sigma_{xx}^{2} + A_2\sigma_{xx}^{1} + A_1\sigma_{xx}^{0} + A_0\sigma_{xx}^{-1}$. Thus, the slope of $\frac{\Delta E^{2\omega}}{(E_{\parallel})^2 B}$ versus σ_{xx}^{3} corresponds to A_4, and the intercept corresponds approximately to A_1. As shown in Fig. S9a, A_1 ~ 0.056 × $10^{-7}AV^{-2}T^{-1}S^{-1}cm^{1}$ for $\chi_{\perp}^{2\omega}$.

Using the same method, we obtain the parameters for $\chi_{\parallel}^{2\omega}$: A_4 ~ 0.38 × 10^{-16} $AV^{-2}T^{-1}\cdot S^{-4}cm^{4}$, A_0 ~ 0.064 × 10^{-4} $AV^{-2}T^{-1}$, and A_1 ~ 0.031 × 10^{-7} $AV^{-2}T^{-1}S^{-1}cm^{1}$, as shown in Figure S8h and Figure S9b. The small magnitudes of A_0 and A_1 indicate that the contributions from σ_{xx}^{0} and σ_{xx}^{1} are negligible.

Using the previously determined A_4, A_1, and A_0 values, we then performed Poly4 fitting for both $\chi_{\perp}^{2\omega}$ and $\chi_{\parallel}^{2\omega}$ to extract the coefficients corresponding to σ_{xx}^2 and σ_{xx}^3, as shown in Fig. S10.

To examine whether σ_{xx}^2 makes a substantial contribution to $\chi^{2\omega}$, we conducted Poly4 fitting under three different conditions:

Type I (Fig. S10a, d): fixing A_4, A_1, and A_0, fitting A_3 and A_2;

Type II (Fig. S10b, e): fixing A_4, A_1, and A_0, with $A_3 = 0$, fitting A_2;

Type III (Fig. S10c, f): setting $A_3 = A_2 = A_1 = A_0 = 0$ and fitting only A_4.

As shown in Fig. S10, all three fitting schemes yield excellent agreement with the measured $\chi^{2\omega}$, with both the R-Square (COD) and Adj. R-Square close to unity. Moreover, for the Type I and II fitting schemes, the contributions from the lower-order σ_{xx}^n terms (*n* = 0-3) largely cancel each other (Table S1), indicating that the inclusion of these lower-order terms does not provide meaningful additional information for Device #1. Consistently, all fitting results demonstrate that the contributions from σ_{xx}^n terms with *n* = 0 to 3 are negligible compared to the quartic scaling. Therefore, the quartic term (σ_{xx}^4) overwhelmingly dominates the magnitude of $\chi_{\perp}^{2\omega}$.

Table S1. Fourth-order polynomial (Poly4) fitting results for three fitting schemes

		A_4 (10^{-16} AV^{-2}T^{-1} ·S^{-4}cm^4)	A_3 (10^{-13} AV^{-2}T^{-1} ·S^{-4}cm^4)	A_2 (10^{-10} AV^{-2}T^{-1} ·S^{-4}cm^4)	A_1 (10^{-7} AV^{-2}T^{-1} ·S^{-4}cm^4)	A_0 (10^{-4} AV^{-2}T^{-1})
$\chi_{\perp}^{2\omega}$	Type I	0.33	-0.08 $\pm$ 0.02	0.04 $\pm$ 0.03	0.056	0.017
	Type II	0.33	0	-0.03 $\pm$ 0.02	0.056	0.017
	Type III	0.33 $\pm$ 0.004	0	0	0	0
$\chi_{\parallel}^{2\omega}$	Type I	0.38	-0.07 $\pm$ 0.03	-0.06 $\pm$ 0.05	0.031	0.064
	Type II	0.38	0	-0.07 $\pm$ 0.03	0.031	0.064
	Type III	0.38 $\pm$ 0.006	0	0	0	0

In conclusion, the NRMT arising from the Zeeman coupling mechanism in Device #1 is negligible, and the quartic scaling mechanism (LSK) is identified as the primary origin.

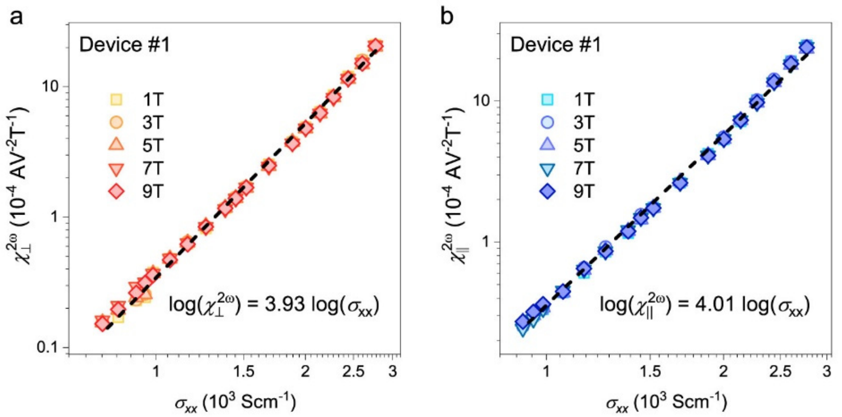

Supplementary Figure S7 | Logarithmic plots of $\chi_{\perp}^{2\omega}$ and $\chi_{\parallel}^{2\omega}$ versus σ_{xx} for Device #1. a, $\chi_{\perp}^{2\omega}$ as a function of σ_{xx} plotted on a logarithmic scale. **b**, $\chi_{\parallel}^{2\omega}$ as a function of σ_{xx} plotted on a logarithmic scale.

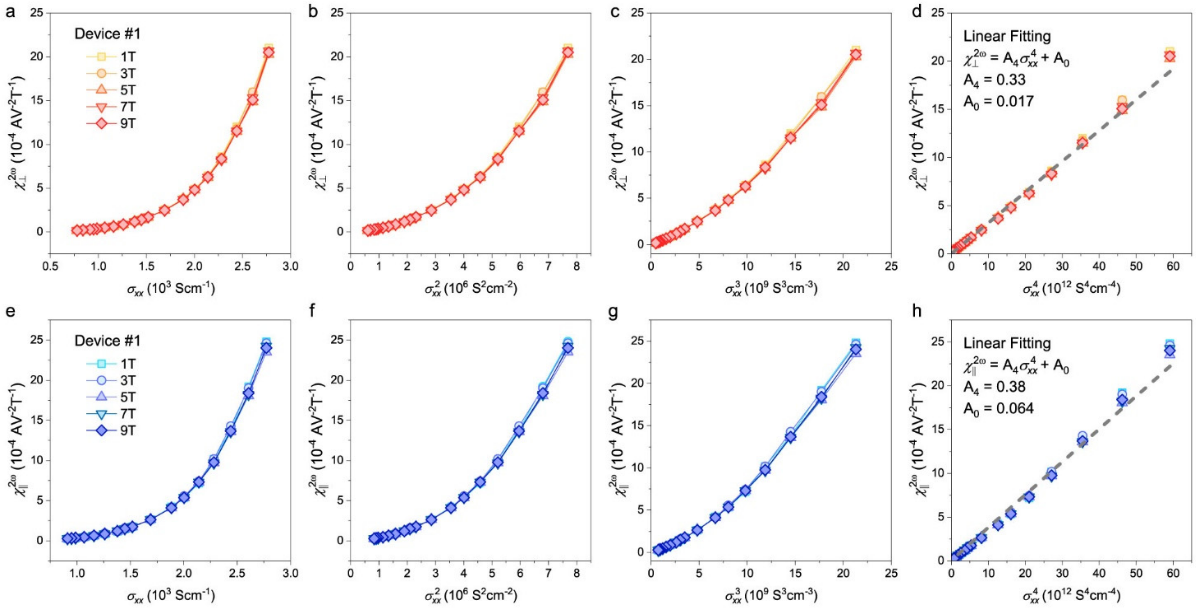

Supplementary Figure S8 | Scaling behavior of $\chi_{\perp}^{2\omega}$ and $\chi_{\parallel}^{2\omega}$ as functions of σ_{xx}^{n} (n = 1 to 4) for Device #1. a-d, $\chi_{\perp}^{2\omega}$ as a function of σ_{xx}^{n} (n = 1 to 4). **e-h**, $\chi_{\parallel}^{2\omega}$ as a function of σ_{xx}^{n} (n = 1 to 4). For $\chi_{\perp}^{2\omega}$, A_4 is ~ 0.33 × 10^{-16} $AV^{-2}T^{-1}\cdot S^{-4}cm^{4}$, A_0 is ~ 0.017 × 10^{-4} $AV^{-2}T^{-1}$; For $\chi_{\parallel}^{2\omega}$, A_4 is ~ 0.38 × 10^{-16} $AV^{-2}T^{-1}\cdot S^{-4}cm^{4}$, A_0 is ~ 0.064 × 10^{-4} $AV^{-2}T^{-1}$.

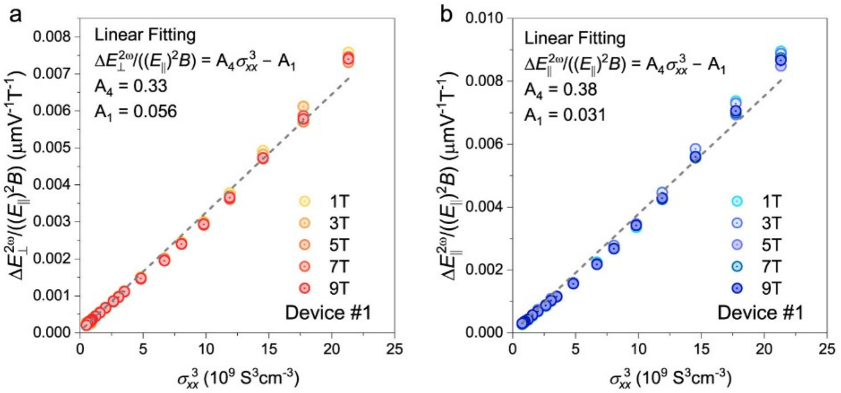

Supplementary Figure S9 | Linear fitting of $\frac{\Delta E_{\perp}^{2\omega}}{(E_{\parallel})^2 B}$ and $\frac{\Delta E_{\parallel}^{2\omega}}{(E_{\parallel})^2 B}$ versus σ_{xx}^3 of Device #1 to get A_1. **a**, For $\frac{\Delta E_{\perp}^{2\omega}}{(E_{\parallel})^2 B}$, A_4 ~ 0.33 × 10^{-16} AV^{-2}T^{-1}·S^{-4}cm^4, A_1 ~ 0.056 × 10^{-7}AV^{-2}T^{-1}S^{-1}cm^1. **b**, For $\frac{\Delta E_{\parallel}^{2\omega}}{(E_{\parallel})^2 B}$, A_4 ~ 0.38 × 10^{-16} AV^{-2}T^{-1}·S^{-4}cm^4, A_1 ~ 0.031 × 10^{-7}AV^{-2}T^{-1}S^{-1}cm^1.

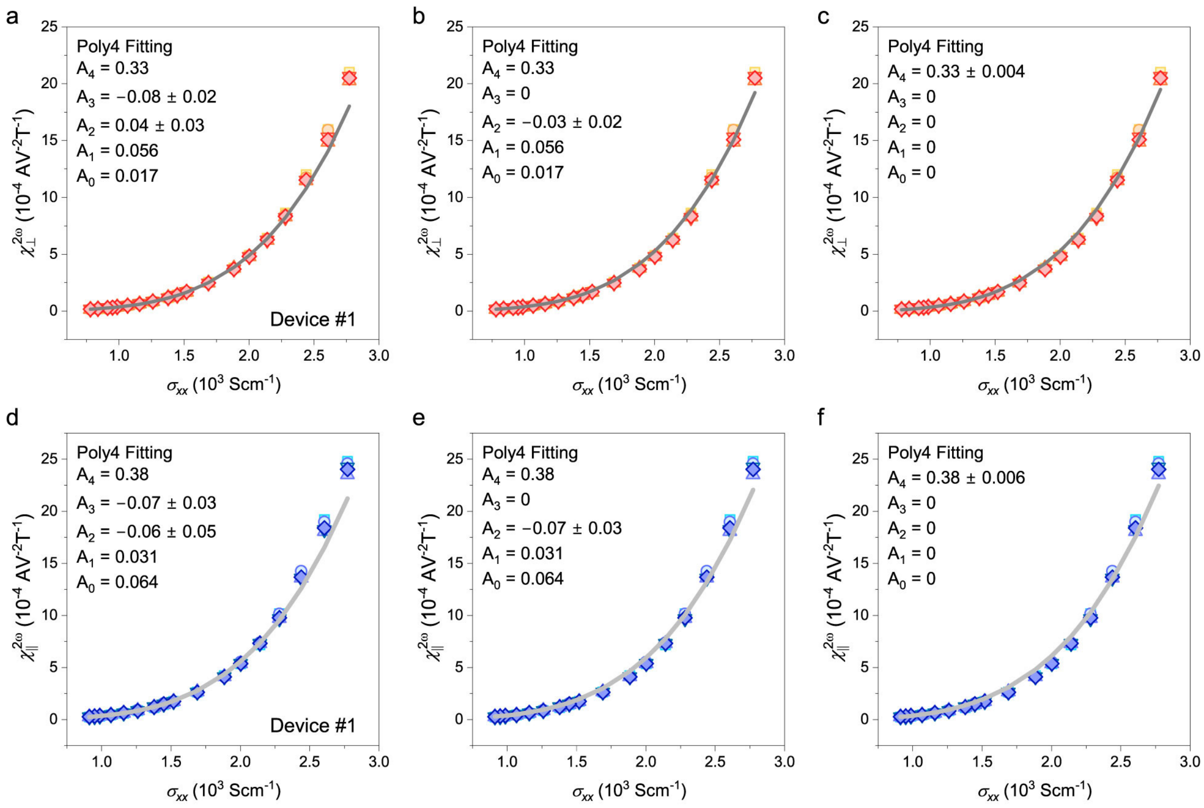

Supplementary Figure S10 | Fourth-order polynomial (Poly4) fits to $\chi_{\perp}^{2\omega}$ and $\chi_{\parallel}^{2\omega}$ for Device #1. Gray lines show the fitting results, and the fitted parameters are listed in the corresponding panels. **a**, **d**, Poly4 fits with A_4, A_1, and A_0 fixed (Type I) for $\chi_{\perp}^{2\omega}$ (**a**), and $\chi_{\parallel}^{2\omega}$ (**d**). **b**, **e**, Poly4 fits with A_4, A_1, A_0 fixed and $A_3 = 0$ (Type II) for $\chi_{\perp}^{2\omega}$ (**b**), and $\chi_{\parallel}^{2\omega}$ (**e**). **c**, **f**, Poly4 fits with $A_3 = A_2 = A_1 = A_0 = 0$ (Type III) for $\chi_{\perp}^{2\omega}$ (**c**), and $\chi_{\parallel}^{2\omega}$ (**f**).

S4. Additional data for BiTeBr Devices #4 to #10

To investigate the crossover from our observed LSK mechanism (σ_{xx}^4 scaling) in high-mobility BiTeBr samples to the previously reported Zeeman-coupling mechanism (σ_{xx}^2 scaling) in BiTeBr with lower mobilities, and to identify whether mobility is the key parameter that determines the dominant origin of NRMT, we fabricated a series of devices (Device #1–#10) spanning a wide mobility range. Before measuring the NRMT response, we first verify their temperature variation of carrier density and selected devices exhibiting only small carrier-density variations for the scaling-law analysis. The temperature-dependent carrier densities of all relevant devices are summarized in Table S2. The detailed data for Devices #4–#10 are provided in Supplementary Figs S11–S17 for reference.

Table S2. The temperature variation of the carrier density of BiTeBr devices

Device	Thickness (nm)	*n* (10^{18} cm^{-3})	Temperature range	Temperature variation
#1	15	10.0-10.7	40-300K	6.5%
#4	104	16.5-17.3	40-240K	4.6%
#5	116.5	31.1-33.6	40-200K	7.4%
#6	32.5	14.2-14.9	30-300K	4.7%
#7	40	6.9-7.3	40-280K	5.5%
#8	133.6	7.3-7.7	40-300K	5.2%
#9	45	7.8-8.1	40-280K	3.7%
#10	45	15.9-16.9	30-300K	5.9%

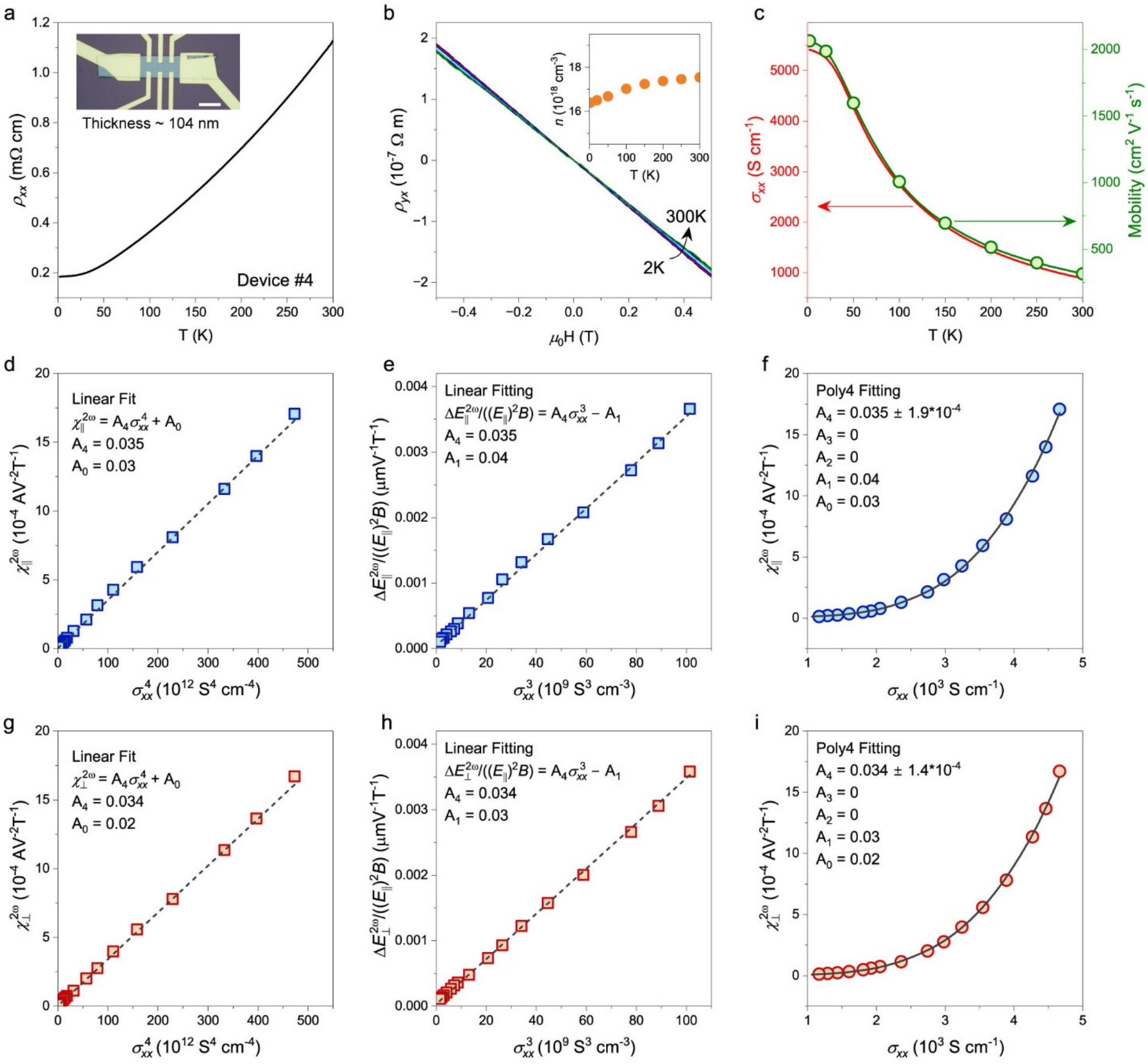

Supplementary Figure S11 | The basic characteritics and the longitudinal NRMT response of Device #4 measured under 7 Tesla, from 40 to 240K. a, The temperature dependence of reisistivity. Insert is the opitcal image of Device #4, scale bar 10 μm. **b**, Hall resistivity ρ_{yx} measured at difference temperature. Insert is the carrier desnity obtained from Hall resistivity. **c**, longitudinal conductivity (left) and mobility (right) of Device #4. **d**, Linear fitting of $\chi_{\parallel}^{2\omega}$ versus σ_{xx}^4 to get A_4 ~ 0.035 × 10^{-16}AV^{-2}T^{-1}·S^{-4}cm^4, A_0 is ~ 0.03 × 10^{-4}AV^{-2}T^{-1}. **e**, Linear fitting of $\frac{\Delta E_{\parallel}^{2\omega}}{(E_{\parallel})^2 B}$ versus σ_{xx}^3 to get A_1 ~ 0.04 × 10^{-7}AV^{-2}T^{-1}S^{-1}cm^1. **f**, Poly4 fitting of $\chi_{\parallel}^{2\omega}$ with fixing A_1, A_0 and setting $A_3 = A_2 = 0$(Type III). **g, h, i**, Scaling analysis of $\chi_{\perp}^{2\omega}$ to get A_4 ~ 0.034 × 10^{-16}AV^{-2}T^{-1}·S^{-4}cm^4, A_1 ~ 0.03 × 10^{-7}AV^{-2}T^{-1}S^{-1}cm^1, and A_0 is ~ 0.02 × 10^{-4}AV^{-2}T^{-1}.

The scaling law of NRMT responses observed in Device #4 (mobility ~ 2064 cm^{2}V^{-1}s^{-1} at 2K) are studied by using the same method described in Supplementary Information S3, as shown in Figure S11. The σ_{xx}^4 contribution is dominant in Device #4 , therefore the LSK mechnism is the main origin. Moreover, in Device

#4 the value of A_4 is ~ 0.035 × 10^{-16}AV^{-2}T^{-1}·S^{-4}cm^{4}, smaller than Device #1, thus the contribution from σ_{xx}^{1} and σ_{xx}^{0} cannot be ignored in high temperatures.

The Data of Device #5 (Mobility ~ 1931 cm^{2}V^{-1}s^{-1} at 2K) are presented in Figure S12. Because the longitudinal ($\chi_{\parallel}^{2\omega}$) and transverse ($\chi_{\perp}^{2\omega}$) NRMT responses are nearly identical, we show only $\chi_{\parallel}^{2\omega}$ here. The NRMT response of Device #5 is dominate by σ_{xx}^{4} contribution (LSK mechanism), and the σ_{xx}^{1} and σ_{xx}^{0} term also contributed at higher temperatures.

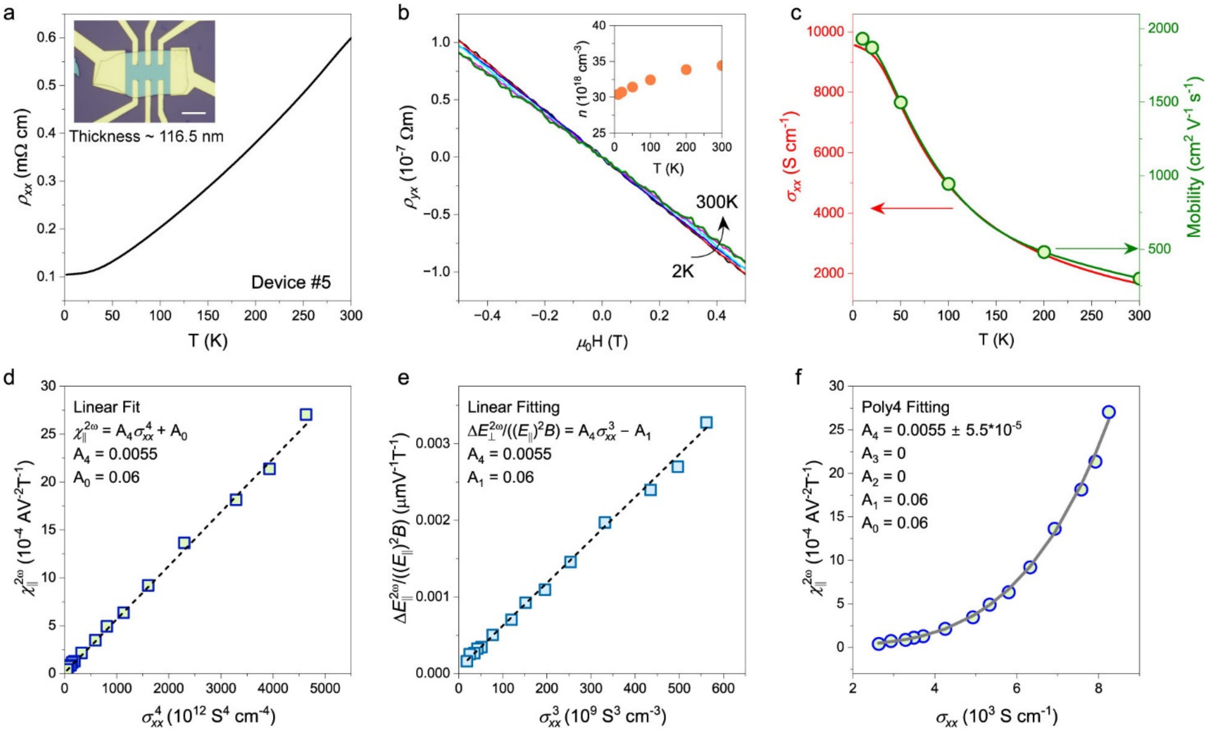

Supplementary Figure S12 | The basic characteritics and the longitudinal NRMT response measured under 7 Tesla, ranging from 40 to 200K of Device #5. a, The temperature dependence of reisistivity. Insert is the opitcal image, scale bar 10 μm. **b**, Hall resistivity ρ_{yx} measured at difference temperature. Insert is the carrier desnity obtained from Hall resistivity. **c**, longitudinal conductivity (left) and mobility (right). **d**, Scaling behavior of $\chi_{\parallel}^{2\omega}$ as functions of σ_{xx}^{4}, A_4 ~ 0.0055 × 10^{-16}AV^{-2}T^{-1}·S^{-4}cm^{4}, A_0 is ~ 0.06 × 10^{-4}AV^{-2}T^{-1}. **e**, Linear fitting of $\frac{\Delta E_{\parallel}^{2\omega}}{(E_{\parallel})^2 B}$ versus σ_{xx}^{3} to get A_1, ~ 0.06 × 10^{-7}AV^{-2}T^{-1}S^{-1}cm^{1}. **f**, Poly4 fitting of $\chi_{\parallel}^{2\omega}$ with setting $A_3 = A_2 = 0$, and fixing A_1, A_0.

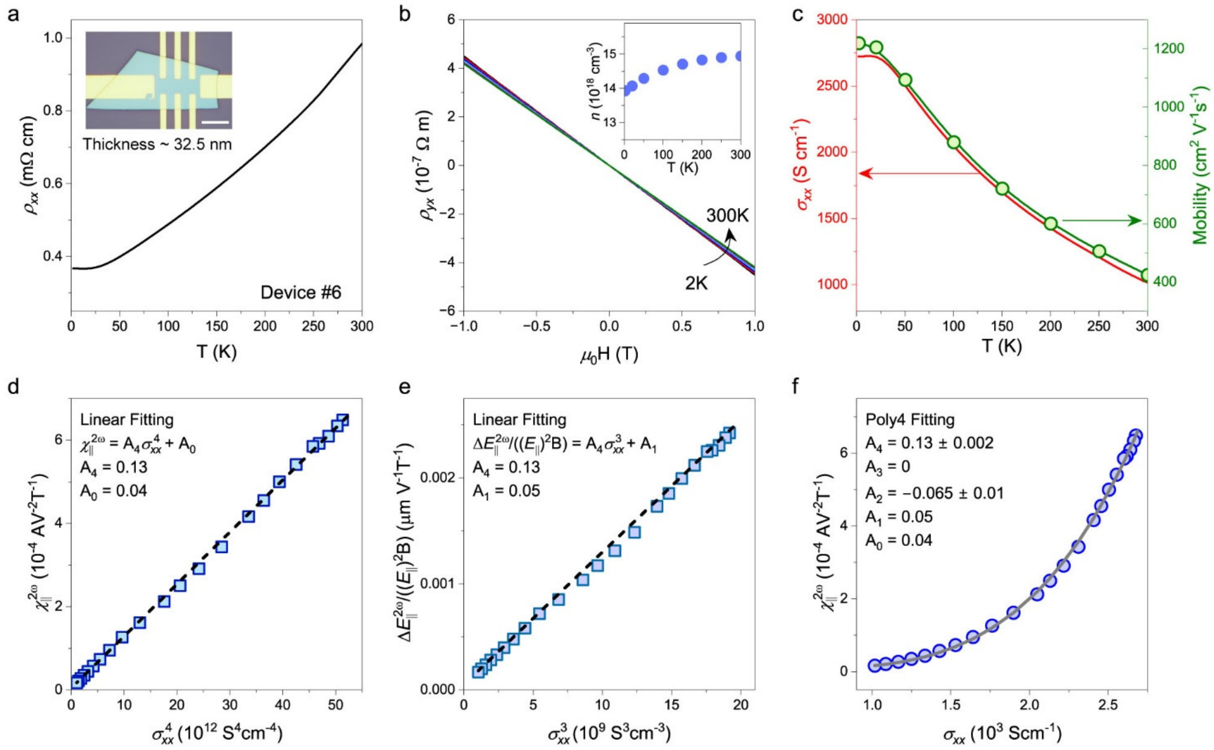

Supplementary Figure S13 | The basic characteritics and the longitudinal NRMT response measured under 7 Tesla, ranging from 30 to 300K of Device #6. **a-c**, Resistivity (**a**), Hall resistivity ρ_{yx} and carrier desnity (**b**), longitudinal conductivity and mobility (**c**) of Device #6. Insert in (**a**) is the opitcal image of Device #6, scale bar 10 μm. **d**, Scaling behavior of $\chi_{\parallel}^{2\omega}$ as functions of σ_{xx}^4, A_4 ~ 0.13×10^{-16} AV^{-2}T^{-1}·S^{-4}cm^4, A_0 is ~ 0.04×10^{-4} AV^{-2}T^{-1}. **e**, Linear fitting of $\frac{\Delta E_{\parallel}^{2\omega}}{(E_{\parallel})^2 B}$ versus σ_{xx}^3 to get A_1, ~ 0.05×10^{-7} AV^{-2}T^{-1}S^{-1}cm^1. **f**, $\chi_{\parallel}^{2\omega}$ as functions of σ_{xx} and the Poly4 fititng with fixing A_3, A_1, A_0, and the fitting results A_4 ~ 0.13×10^{-16} AV^{-2}T^{-1}·S^{-4}cm^4, A_2 ~ -0.065×10^{-10} AV^{-2}T^{-1}·S^{-2}cm^2.

The Data for Device #6 (Mobility$_{2K}$ = 1245.4 cm^{2}V^{-1}s^{-1}) are shown in Figure S13. The NRMT response scales approximatively linearly with σ_{xx}^4, as illustrated in Figure S14d. The coefficients A_1 and A_0 were obtained using the same method described above. With the determined A_1 and A_0 (and $A_3 = 0$), we performed Poly4 fitting to extract the coefficients associated with the σ_{xx}^4 and σ_{xx}^2 terms, yielding A_4 ~ 0.13×10^{-16} AV^{-2}T^{-1}·S^{-4}cm^4 and A_2 ~ -0.065×10^{-10} AV^{-2}T^{-1}·S^{-2}cm^2. Although A_4 is larger than A_2, their magnitudes are not drastically different. Thus, while the σ_{xx}^4 term remains dominant in Device #6, the σ_{xx}^2 contribution cannot be neglected, particularly at higher temperatures. Therefore, the LSK mechanism dominates the NRMT response in Device #6, but the Zeeman-coupling mechanism still provides a minor contribution. Based on these results, the mobility of Device #6 (1220 to 425 cm^{2}V^{-1}s^{-1}) may lie near the boundary separating the LSK-dominated regime from the LSK and Zeeman-coupling both contributed regime.

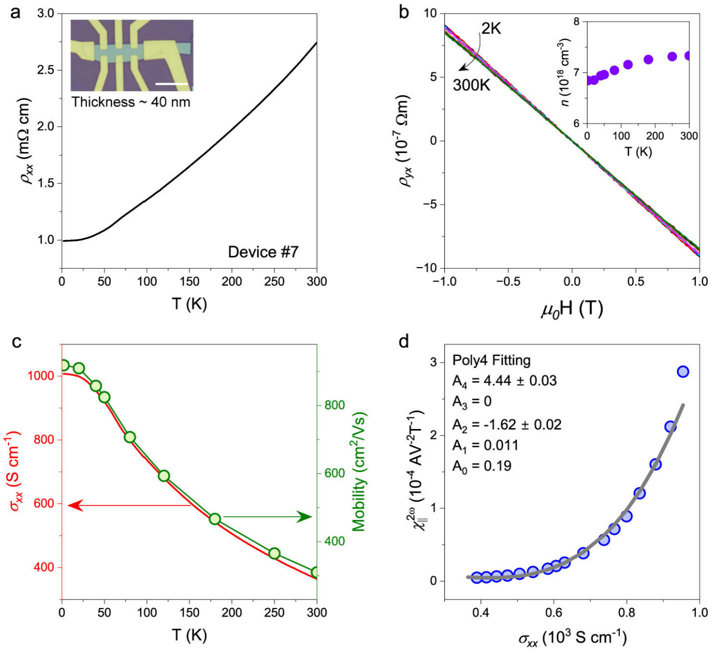

Supplementary Figure S14 | The basic characteritics and the longitudinal NRMT response measured under 7 Tesla, raning from 40 to 280K of Device #7. **a-c**, Resistivity (**a**), Hall resistivity ρ_{yx} at difference temperature and carrier desnity obtained from Hall resistivity (**b**), longitudinal conductivity and mobility (**c**). Insert in (**a**) is the opitcal image, scale bar 10 μm. **d**, $\chi_{\parallel}^{2\omega}$ as functions of σ_{xx} and the Poly4 fititng with fixing $A_3 = 0$, $A_1 \sim 0.011 \times 10^{-7}\mathrm{AV^{-2}T^{-1}S^{-1}cm^{1}}$, $A_0 \sim 0.19 \times 10^{-4}\mathrm{AV^{-2}T^{-1}}$, and the fitting results $A_4 \sim 4.44 \times 10^{-16}\mathrm{AV^{-2}T^{-1}\cdot S^{-4}cm^{4}}$, $A_2 \sim -1.62 \times 10^{-10}\mathrm{AV^{-2}T^{-1}\cdot S^{-2}cm^{2}}$.

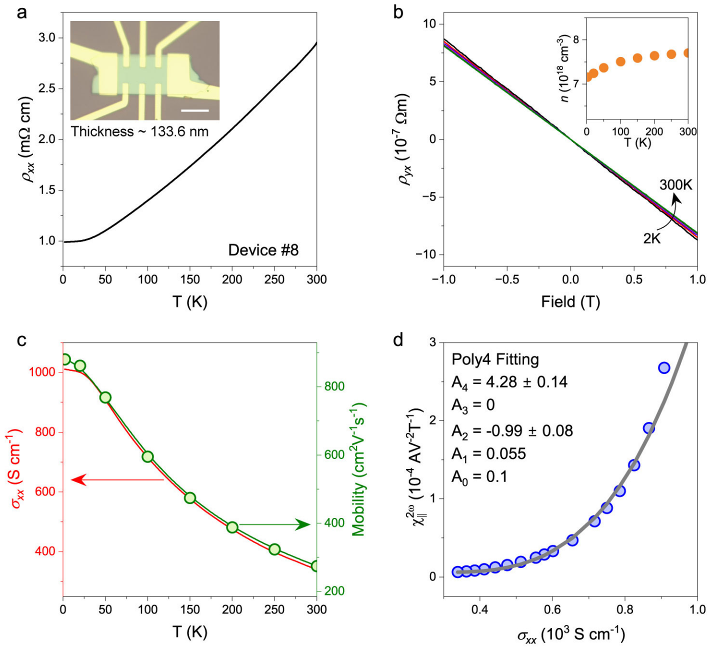

Supplementary Figure S15 | The basic characteritics and the longitudinal NRMT response measured under 7 Tesla, ranging from 40 to 280K of Device #8. **a-c**, Resistivity (**a**), Hall resistivity ρ_{yx} and carrier desnity (**b**), longitudinal conductivity and mobility (**c**). Insert in (**a**) is the opitcal image, scale bar 10 μm. **d**, $\chi_{\parallel}^{2\omega}$ as functions of σ_{xx} and the Poly4 fititng with fixing $A_3 = 0$, $A_1 \sim 0.055 \times 10^{-7}\mathrm{AV^{-2}T^{-1}S^{-1}cm^{1}}$, $A_0 \sim 0.1 \times 10^{-4}\mathrm{AV^{-2}T^{-1}}$, and the fitting results $A_4 \sim 4.28 \times 10^{-16}\mathrm{AV^{-2}T^{-1}\cdot S^{-4}cm^{4}}$, $A_2 \sim -0.99 \times 10^{-10}\mathrm{AV^{-2}T^{-1}\cdot S^{-2}cm^{2}}$.

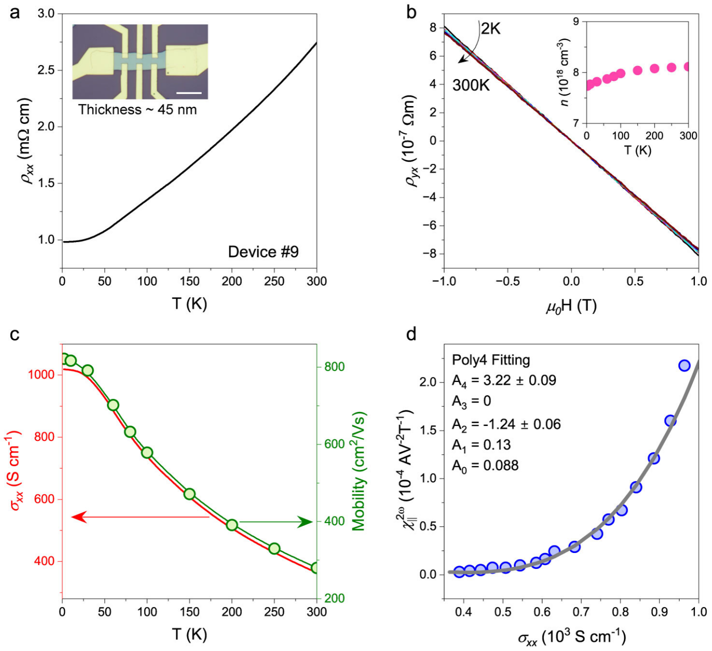

Supplementary Figure S16 | The basic characteritics and the longitudinal NRMT response measured under 7 Tesla, raning from 40 to 280K of Device #9. **a-c**, Resistivity (**a**), Hall resistivity ρ_{yx} at difference temperature and carrier desnity obtained from Hall resistivity (**b**), longitudinal conductivity and mobility (**c**). Insert in (**a**) is the opitcal image, scale bar 10 μm. **d**, $\chi_{\parallel}^{2\omega}$ as functions of σ_{xx} and the Poly4 fititng with fixing $A_3 = 0$, $A_1 \sim 0.13 \times 10^{-7}$AV^{-2}T^{-1}S^{-1}cm^{1}, $A_0 \sim 0.088 \times 10^{-4}$AV^{-2}T^{-1}, and the fitting results $A_4 \sim 3.22 \times 10^{-16}$AV^{-2}T^{-1}·S^{-4}cm^{4}, $A_2 \sim -1.24 \times 10^{-10}$AV^{-2}T^{-1}·S^{-2}cm^{2}.

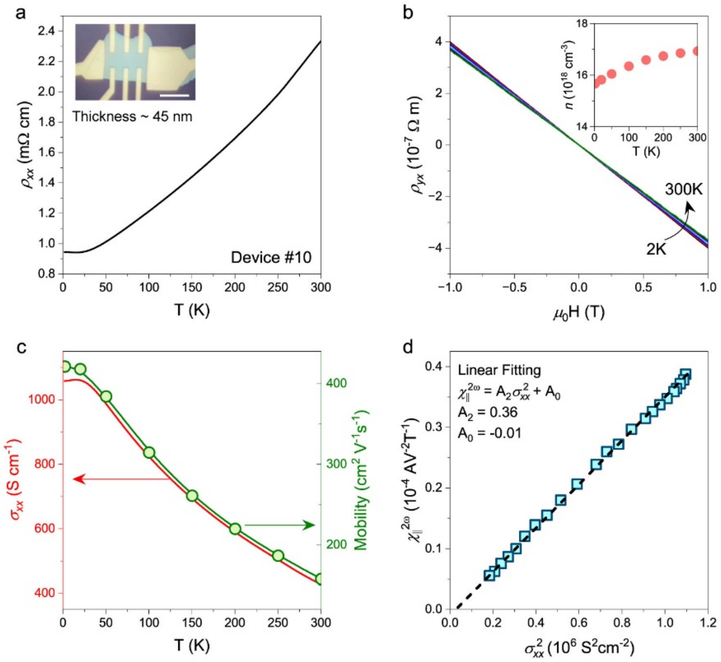

Supplementary Figure S17 | The basic characteritics and the longitudinal NRMT response measured under 7 Tesla, ranging from 30 to 300K of Device #10. **a-c**, Resistivity (**a**), Hall resistivity ρ_{yx} and carrier desnity (**b**), longitudinal conductivity and mobility (**c**). Insert in (**a**) is the opitcal image, scale bar 10 μm. **d**, Scaling behavior of $\chi_{\parallel}^{2\omega}$ as functions of σ_{xx}^2, $A_2 \sim -0.36 \times 10^{-10}$AV^{-2}T^{-1}·S^{-4}cm^{4}, $A_0 \sim 0.01 \times 10^{-4}$AV^{-2}T^{-1}.

The Data of Device #7, #8, #9 are presented in Figures S14-S16. These devices exhibit relatively low mobilities (~900 $cm^2V^{-1}s^{-1}$ at 2K). Their NRMT responses do not scale linearly with σ_{xx}^4, indicating that the σ_{xx}^2 term may also make a substantial contribution. We also applied a Poly4 fitting to extract the coefficients A_4 to A_0. We first obtained approximate values for A_1 and A_0 using the same method as before. By setting $A_3 = 0$ and fixing A_1 and A_0, we then extracted A_4 and A_2 using the Poly4 fit, as shown in Figure S14d, S15d, S16d. The fitting results show that both σ_{xx}^4 and σ_{xx}^2 contribute significantly to the NRMT response. However, because the A_4 and A_2 have opposite signs, the σ_{xx}^4 and σ_{xx}^2 terms provide opposite contributions. This indicates that the LSK mechanism and the Zeeman-coupling mechanism contribute with opposite signs to the NRMT response in BiTeBr devices.

The Data of Device #10 (mobility ~ 432 $cm^2V^{-1}s^{-1}$ at 2K) are presented in Figure S17. Unlike the previous devices, the NRMT response of Device #10 scales almost linearly with σ_{xx}^2. We obtained the coefficient A_2 and A_0 from a linear fit of $\chi_{\parallel}^{2\omega}$ versus σ_{xx}^2, yielding $A_2 = 0.36$, $A_0 = -0.01$. Because the σ_{xx}^2 term contributes with with the opposite sign to the σ_{xx}^4 and σ_{xx}^0 term, and in our convention the σ_{xx}^4 contribution is taken as positive, thus the coefficient are $A_2 \sim -0.36 \times 10^{-10} AV^{-2}T^{-1} \cdot S^{-4} cm^4$, and $A_0 \sim 0.01 \times 10^{-4} AV^{-2}T^{-1}$. Based on the observed scaling behavior of NRMT in Device #10, the mobility of Device #10 (421 to 158 $cm^2V^{-1}s^{-1}$) places it within the Zeeman-coupling-dominated regime.

The basic characteristics, together with the corresponding scaling behavior and dominant NRMT mechanisms of all measured devices, are summarized in Table S3. Devices with relatively high mobility (~2000 $cm^2V^{-1}s^{-1}$ at 2K), such as Devices #1, #4, and #5, exhibit NRMT dominated by the σ_{xx}^4 term. By contrast, low-mobility devices (~420 $cm^2V^{-1}s^{-1}$ at 2K), such as Device #10, show NRMT arising almost entirely from the σ_{xx}^2 contribution, consistent with previous Nature Physics work (*Nat. Phys.* 13, 578-583 (2017)). Devices with intermediate mobilities (~900 $cm^2V^{-1}s^{-1}$ at 2K), including Devices #7, #8, and #9, display mixed behavior in which both σ_{xx}^4 and σ_{xx}^2 terms contribute. Device #6 with an intermediate but higher mobility (~1220 $cm^2V^{-1}s^{-1}$ at 2K), is dominated by the σ_{xx}^4 (LSK) mechanism but shows a non-negligible σ_{xx}^2 contribution at elevated temperatures, placing it near the crossover between purely LSK-dominated and mixed-mechanism regimes.

Table S3. The basic characteristics of BiTeBr devices, their dominated σ_{xx} contribution and mechnism

Device	Thickness (nm)	Carrier density n (10^{18} cm^{-3})	Mobility (cm^{2}V^{-1}s^{-1})	τ @ 40K (ps)	Dominate σ_{xx} contribution	Dominate mechanism
#1	15	10.0-10.7	1934-454	0.15	σ_{xx}^4	LSK
#4	104	16.5-17.3	2064-431	0.15	σ_{xx}^4	LSK
#5	116.5	31.1-33.6	1931-482	0.14	σ_{xx}^4	LSK
#6	32.5	14.2-14.9	1220-425	0.096	σ_{xx}^4, σ_{xx}^2	LSK and ZC
#7	40	6.9-7.3	919-310	0.073	σ_{xx}^4, σ_{xx}^2	LSK and ZC
#8	133.6	7.3-7.7	880-274	0.068	σ_{xx}^4, σ_{xx}^2	LSK and ZC
#9	45	7.8-8.1	822-280	0.065	σ_{xx}^4, σ_{xx}^2	LSK and ZC
#10	45	15.9-16.9	421-158	0.034	σ_{xx}^2	ZC

S5. First-principles band structures calculations of BiTeBr

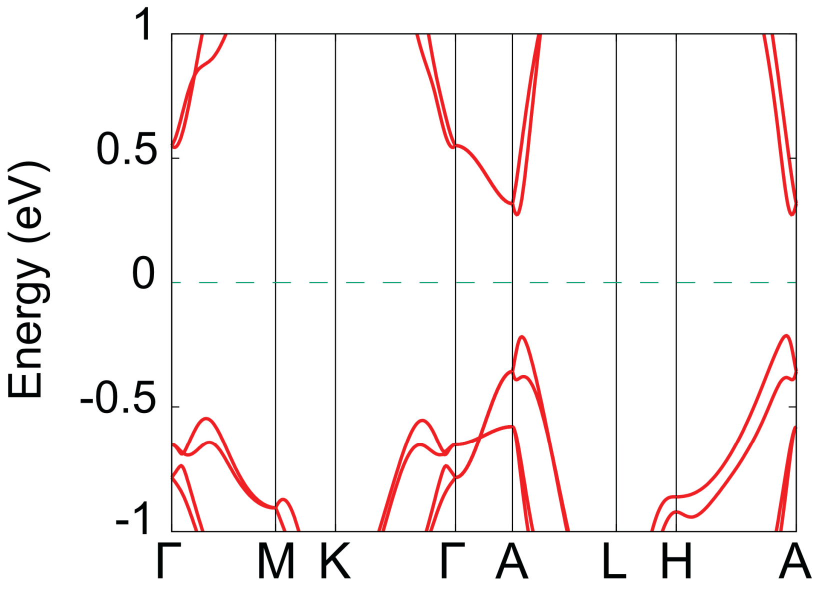

Supplementary Figure S18 | Band structure of BiTeBr. Band structure of BiTeBr calculated by first-principles calculations along the high-symmetry *k*-path Γ-M-K-Γ-A-L-H-A in the Brillouin zone. Calculations were performed using the Vienna Ab initio Simulation Package with spin–orbit coupling included. The Fermi level is set to 0 eV, indicated by the dashed line.
