# Supplementary material for: Lorentz skew scattering nonreciprocal magneto-transport
Source: Nat Commun. 2026 May 8;17:6221. doi: 10.1038/s41467-026-71789-y (PMC13369428; doi:10.1038/s41467-026-71789-y)
Supplement: Supplementary file 2 — Transparent Peer Review file [file 41467_2026_71789_MOESM2_ESM.pdf]

nature portfolio

Peer Review File

Lorentz Skew Scattering Nonreciprocal Magneto-Transport

Corresponding Author: Professor Weibo Gao

This manuscript has been previously reviewed at another journal. This document only contains information relating to versions considered at Nature Communications.

This file contains all reviewer reports in order by version, followed by all author rebuttals in order by version.

Version 0:

Reviewer comments:

Reviewer #1

(Remarks to the Author)
In the revised manuscript, the authors supplemented their claims by conducting additional experiments and analyses. Regarding the points I raised, the discussion has been improved, and I feel the arguments have gained a certain degree of persuasiveness. I have no further comments to add.

Reviewer #4

(Remarks to the Author)
After reading reviewers' comments and authors' responses as well as the revised version of the manuscript, I am confident that it is of high quality. I fully support the publication of the revised manuscript in Nature Communications.

Open Access This Peer Review File is licensed under a Creative Commons Attribution 4.0 International License, which permits use, sharing, adaptation, distribution and reproduction in any medium or format, as long as you give appropriate credit to the original author(s) and the source, provide a link to the Creative Commons license, and indicate if changes were

made.
In cases where reviewers are anonymous, credit should be given to 'Anonymous Referee' and the source.
The images or other third party material in this Peer Review File are included in the article's Creative Commons license, unless indicated otherwise in a credit line to the material. If material is not included in the article's Creative Commons license and your intended use is not permitted by statutory regulation or exceeds the permitted use, you will need to obtain permission directly from the copyright holder.
To view a copy of this license, visit https://creativecommons.org/licenses/by/4.0/

Response to Reviewers' comments

We sincerely express our gratitude to all reviewers for dedicating their time to thoroughly evaluating our work. We are glad that Reviewer #1 and Reviewer #4 have recommended publication of our work. In response, we have diligently addressed each question and comment raised by Reviewer #1 and #4. Below, we provide a detailed, point-by-point response.

Reviewer #1 (Remarks to the Author):

‘In the revised manuscript, the authors supplemented their claims by conducting additional experiments and analyses. Regarding the points I raised, the discussion has been improved, and I feel the arguments have gained a certain degree of persuasiveness. I have no further comments to add.’

Response:

We sincerely thank the reviewer for the positive evaluation of our revised manuscript. We are pleased that the additional experiments, analyses, and revisions have addressed the reviewer’s previous concerns and improved the clarity and persuasiveness of the discussion. We greatly appreciate the reviewer’s constructive feedback throughout the review process.

Reviewer #4 (Remarks to the Author):

‘After reading reviewers’ comments and authors’ responses as well as the revised version of the manuscript, I am confident that it is of high quality. I fully support the publication of the revised manuscript in Nature Communications.’

Response:

We sincerely thank the reviewer for the very positive assessment and strong support for the publication of our work in *Nature Communications*. We greatly appreciate the reviewer’s time and thoughtful evaluation during the review process.
